# Molecular phylogenetics and evolutionary history of *Bienertia sinuspersici*: implications for crop improvement in South Asia

**DOI:** 10.3389/fpls.2026.1822164

**Published:** 2026-06-09

**Authors:** Qasim Ali Hashmi, Joonho Park

**Affiliations:** Department of Fine Chemistry, Seoul National University of Science and Technology, Seoul, Republic of Korea

**Keywords:** *Bienertia sinuspersici*, C4 photosynthesis, crop resilience, genetic engineering, salt tolerance, single cell C4 photosynthesis, photosynthetic efficiency, halophytes

## Abstract

This review’s scientific value is to systematically review existing literature on *B. sinuspersici* ensuring a structure of exploiting such genetic adapt reactions to counter climate change impacts on agriculture. This work highlights the need for such interdisciplinarity to bring such discoveries into fruition of improved, salinity and drought, tolerant crops, providing novel tactics toward food production from saline and arid landscapes. This paper argues that *Bienertia sinuspersici* has revolutionary genes for enhancing crop varieties and global food security in regions that are affected by climate change, synthesises current knowledge of the molecular phylogenetics, evolution, adaptive physiology, and translational potential of *Bienertia sinuspersici*, with particular emphasis on its application to crop improvement. As one of the few known plants performing fully functional single-cell C_4_ photosynthesis in a halophytic context, *B. sinuspersici* provides a valuable genetic reservoir for enhancing salt and drought tolerance in staple cereals such as rice (*Oryza sativa*) and wheat (*Triticum aestivum*). We examine the physiological and molecular traits that enable *B. sinuspersici* to thrive under extreme abiotic stress including ion homeostasis, osmoprotectant biosynthesis, and photosynthetic efficiency and evaluate the prospects for transferring these traits into glycophytic crops through transgenic approaches and marker-assisted selection. Key findings centre on stress-responsive genes, notably high-affinity potassium transporters (HKT1) and sodium/hydrogen exchangers (NHX1), which are strongly associated with salinity tolerance. Recent advances in CRISPR-Cas9 genome editing and genome-wide association studies (GWAS) further expand the toolkit for introgressing *B. sinuspersici*-derived traits into food crops, with direct implications for global food security. However, significant gaps remain, particularly the absence of multi-year, multi-location field trials validating these traits under realistic agronomic conditions. The principal contribution of this review is a systematic integration of the available literature on *B. sinuspersici*, framed as a roadmap for harnessing its adaptive genetic resources to mitigate the agricultural impacts of climate change. We argue that realizing this potential will require sustained interdisciplinary collaboration spanning molecular biology, plant breeding, and agronomy, and that *B. sinuspersici* offers transformative genetic resources for developing salinity- and drought-tolerant cultivars suited to saline and arid production systems.

## 1 Introduction

Soil salinization and chronic water deficit now threaten an estimated 1.4 billion hectares of agricultural land globally, with South Asia among the worst-affected regions according to Food and Agriculture Organization. Against this backdrop, halophytic plants species that complete their life cycles in high-salinity environments represent an underexplored reservoir of genetic solutions for food-security crops such as rice (*Oryza sativa*) and wheat (*Triticum aestivum*). Among halophytes, *Bienertia sinuspersici* Akhani (Amaranthaceae) stands out for a combination of traits that is unique in the plant kingdom: it performs fully functional C_4_ photosynthesis within a single chlorenchyma cell, without the Kranz anatomy that defines all other known C_4_ lineages (Chuong et al., 2006; Edwards et al., 2004). The single-cell C_4_ system in which peripheral chloroplasts fix CO_2_ via phosphoenolpyruvate carboxylase (PEPC) and central chloroplasts re-fix it via Rubisco and NAD-malic enzyme delivers the carbon-concentrating benefits of conventional C_4_ metabolism while operating entirely within one cell type. Combined with constitutively high expression of ion transporters (HKT1, NHX1, SOS1) and robust osmoprotectant pathways, this physiology allows *B. sinuspersici* to thrive in soils where Na^+^ concentrations far exceed the tolerance limits of even the most stress-resistant crops.

Within the Amaranthaceae, *B. sinuspersici* occupies an evolutionarily isolated position. Molecular phylogenetic analyses using ribosomal, plastid, and mitochondrial markers have confirmed its placement as a distinct lineage within the chenopod clade, diverging from closely related halophytic genera such as *Suaeda* and *Salicornia* approximately 10–15 million years ago a divergence coinciding with late Miocene aridification of Southwest Asia (Berasategui et al., 2023; Rudov et al., 2020). That both *Suaeda* and *Salicornia* employ a conventional two-cell C_4_ pathway underscores the evolutionary novelty of the single-cell mechanism in *B. sinuspersici*, and raises fundamental questions about the minimum genetic requirements for a functional carbon-concentrating mechanism questions with direct implications for engineering C_4_-like traits in C_3_ crops (Sharpe et al., 2020).

Substantial progress has been made in characterizing the biochemistry and cell biology of *B. sinuspersici*. The spatial segregation of peripheral chloroplasts (PEPC, PPDK activity) from central chloroplasts (Rubisco, NAD-ME activity) has been mapped at the protein level, and comparative genomic studies have identified expanded gene families for ion transport and osmolyte biosynthesis relative to other Amaranthaceae members (Mehra et al., 2024; Urbanavičiūtė et al., 2021a). RNA-Seq profiling under saline and drought treatments has revealed a suite of consistently up-regulated stress-responsive genes, most notably *BtHKT1* (a species-specific high-affinity potassium transporter variant), *SERF-1* (a stress-responsive ERF transcription factor absent or lowly expressed in most crops), and an uncharacterized Salt-Responsive Pathway 1 of currently unknown function (Nivedita et al., 2021; Wani et al., 2020). QTL mapping and GWAS have additionally linked specific genomic regions to Na^+^ compartmentalization, water-use efficiency, and osmolyte accumulation.

Despite this progress, no review has yet integrated the evolutionary history of *B. sinuspersici* with a systematic evaluation of its novel genetic resources for crop breeding. Existing literature is either focused on the biochemistry of the C_4_ mechanism in isolation, or on single gene-transfer experiments in model crops; crucially, the species’ taxonomy, geographic diversity, domestication potential, and the novelty of its alleles relative to genes already available in mainstream breeding programs have not been comprehensively assessed. This gap limits the practical utility of the growing body of molecular data for breeders and biotechnologists working in saline-prone South Asian agricultural systems.

The present review addresses this gap with three specific objectives. First, it consolidates and critically evaluates the molecular phylogenetic and evolutionary evidence for *B. sinuspersici* ‘s unique position within the Amaranthaceae and the paleoclimatic forces that shaped its single-cell C_4_ adaptation. Second, it synthesizes genomic, transcriptomic, and genetic mapping data currently dispersed across the literature into a unified account of the molecular architecture underlying C_4_ photosynthesis and abiotic stress tolerance in this species, with explicit identification of novel genes and alleles that are not yet exploited in crop improvement. Third, it evaluates the translational pathways including marker-assisted selection, CRISPR-Cas9 gene editing, and accelerated *de novo* domestication through which these genetic resources could be deployed to improve salinity and drought tolerance in South Asian staple crops.

The review is organised as follows. Section 2 provides an integrated account of the taxonomy, phylogenetic diversity, geographic distribution, economic significance, and domestication status of *B. sinuspersici*. Section 3 examines its molecular phylogenetics and evolutionary history. Section 4 describes the biophysical and molecular mechanisms of single-cell C4 photosynthesis. Section 5 synthesises all genomic, transcriptomic, GWAS, and QTL evidence relevant to the C4 pathway and abiotic stress tolerance, including a priority table of novel alleles for breeders. Section 6 discusses applications for crop improvement, with particular attention to accelerated domestication. Section 7 identifies key research gaps and outlines future directions.

## 2 Taxonomy, ecophysiology, and biological significance of *B. sinuspersici*

### 2.1 Historical placement in Chenopodiaceae

*Bienertia sinuspersici* was originally classified within the family Chenopodiaceae., a predominantly temperate and subtropical family typified by halophytic and xerophytic herbs and subshrubs adapted to saline, alkaline, and arid substrates. Within Chenopodiaceae, the genus Bienertia Bunge ex Boiss. was assigned to subfamily Suaedoideae and tribe Bienertieae a small, morphologically coherent tribe characterised by semi-terete or terete succulent leaves, reduced perianth, and, uniquely among angiosperms, single-cell C4 photosynthesis without Kranz anatomy (Kadereit et al., 2003; Kapralov et al., 2006). The genus was historically treated as monotypic, comprising only *B. cycloptera* Bunge ex Boiss., before the formal description of *B. sinuspersici* as a second species and subsequently *B. kavirense* as a third, expanding the genus to its current three-species circumscription (Xu et al., 2024).

#### 2.1.1 Taxonomic history and reclassification in Amaranthaceae

*Bienertia sinuspersici* is a halophytic species it was classified under the family Chenopodiaceae but the current molecular phylogenetic data places it under the family Amaranthaceae. In the past, the identification of *B. sinuspersici* was mainly done according to its morphological traits, ecological adaptations including its tolerance to saline conditions and its C4 photosynthetic pathway (Berasategui et al., 2023; Xu et al., 2024). It was not long ago that early researchers used its habitat and structure effectively for taxonomy, but the molecular methods are not available now. Placing *B. sinuspersici* into the Amaranthaceae family is quite noteworthy because this family contains other crops such as quinoa (*Chenopodium quinoa*) and spinach (Spinacia oleracea) which have been also recognized to possess stress tolerance mechanisms under different environmental stress conditions (Jamalluddin et al., 2022). However, *B. sinuspersici* can be distinctive based on its unique single-cell C4 photosynthesis pathway, and many researchers and scientists have explored this plant because of its overall interest to biological taxonomy and evolution (Rudov et al., 2020). This uniqueness made its taxonomy highly questionable until advances in molecular genetics and phylogenetics offered some answers into its systematic relationship.

*B. sinuspersici* taxonomical position has been made clear by sequences of ribosomal DNA (rDNA), plastid and mitochondrial DNA. The taxonomical groupings derived earlier from the morphological characteristics were replaced with the help of molecular characteristics; it was confirmed that *B. sinuspersici* is related to the genera such as Salicornia & Suaeda (Park et al., 2020). Its evolutionary relationships are well understood based on analyses of molecular phylogenetics with the help of Bayesian Inference and Maximum Likelihood trees. As evidenced by these studies, *B. sinuspersici* split from its congeners about 10–15 million years ago probably due to changes in climatic conditions that enabled the habitation of saline and arid sites (Rudov et al., 2020).

### 2.2 Geographic distribution and ecological range

*Bienertia sinuspersici* occupies a geographically restricted but ecologically coherent range centred on the arid lowland zones of Southwest Asia, primarily around the Persian Gulf basin and the northern shoreline of the Gulf of Oman (Akhani et al., 2005; Bachman et al., 2024). At the country level, documented occurrence spans Iraq (widespread in desert regions), Iran (coastal lowlands of Khuzestan, Hormozgan, and Bushehr provinces, extending into inland saline flats), Saudi Arabia, the United Arab Emirates, Kuwait, Qatar, and the Sultanate of Oman (Akhani et al., 2005; Bachman et al., 2024) The species was formally described by (Akhani et al., 2005) and its epithet sinuspersici directly references its primary distributional range around the Sinus Persicus (Persian Gulf), underscoring the inseparability of the species’ identity from its geographic origin. Globally, all three confirmed species of the genus Bienertia (*B. sinuspersici*, *B. cycloptera*, and *B. kavirense*) are endemic to Southwest Asia, with the range of *B. sinuspersici* restricted almost entirely to the hot, hyper-arid coastal and sub-coastal lowlands of this region (Rudov et al., 2020).

**Figure 1:**
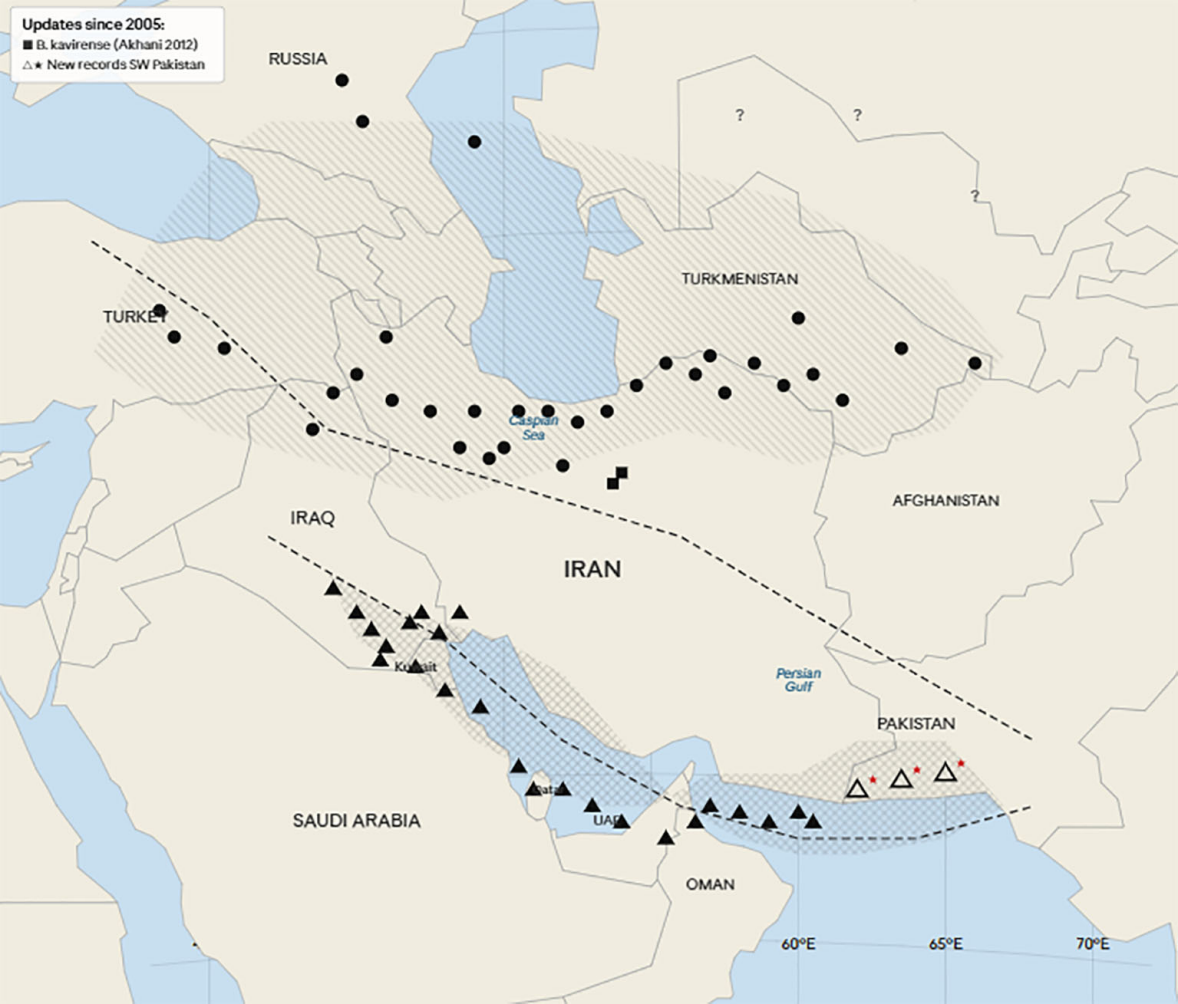
Geographical distribution of *Bienertia sinuspersici* across Southwest and Central Asia. • *B. cycloptera* (temperate/cold deserts, higher latitudes). ▴ *B. sinuspersici* (hot arid zones, Persian Gulf). ◼ *B. kavirense* (Dasht-e Kavir, Iran only 2012). △ = new post-2005 records (SW Pakistan) Record modified from (Akhani et al., 2005).

The geographic distribution of *B. sinuspersici* provides insights in the distribution of the genetic variation and population structure of this species in South Asia. Cross-sectional population genetics based on microsatellite and SNP studies have characterized separate genetically coherent populations adapted to specific level of salinity. Such studies indicate that climatic fluctuations that occurred in the past and geographical factors have impacted on the genetic stock and spread of *B. sinuspersici* (Nukazawa et al., 2023). Now geographical distribution of *Bienertia sinuspersici* extends from the Persian Gulf to the Balochistan regions of Pakistan GBIF.org, 2026 (**Figure 1**). All these studies are source of knowledge for understanding how the mechanisms of genetic exchange and adaptability may work under various circumstances. They also emphasize on the need to protect *B. sinuspersici* ‘s genetic variation to retain the possibility of using biotic potential for improvement of improved cultivars (Jamalluddin et al., 2022).

The species is found predominantly in the deserts and the saline regions of Middle Eastern and South Asian areas. Genetic variation within populations is therefore relatively high for a species that inhabits such strict habitats, suggesting that the species has faced multiple selective forces over its evolution. Researchers have established that *B. sinuspersici* in South Asia have distinct genetic strains that imply temporal separation and narrow ecological specialization. This structural population is in agreement with the model of-genetic differentiation due to environmental factors, especially where there is a difference in the salinity and the composition of the soil (Gai et al., 2021). Reproductive potentials have also been cited in relation to genetic variation in *B. sinuspersici*. This plant reproduces both sexually through seed and asexually through vegetative processes to ensure that the species stays genetically diverse because of the conditions it faces in its environment. This diversification is essential for its existence since it is a source of genes out of which a trait adapted to a change in environment can evolve.

#### 2.2.1 Chloroplast genome evidence and affinities within Amaranthaceae

Comparative chloroplast genome analysis provides additional resolution of *B. sinuspersici* ‘s affinities within Amaranthaceae. At 70% sequence identity, *B. sinuspersici* is most closely related to *Beta vulgaris L*. (sugar beet, subfamily Betoideae), with minor differences in gene arrangement, including variation in ycf1, ycf15, and the absence of psbB in Beta. The chloroplast genome GC content of *B. sinuspersici* (36.6%) is identical to that of Haloxylon persicum and H. ammodendron (subfamily Salsoloideae), with 107 tandem repeats predominantly located in intergenic regions (Park, 2018). These chloroplast data complement nuclear and multi-locus molecular evidence confirming *B. sinuspersici* ‘s placement as a highly specialised chenopod lineage within the broadly circumscribed Amaranthaceae, the largest C4 eudicot family, in which C4 photosynthesis is estimated to have evolved independently at least 15 times (Kadereit et al., 2003; Siadjeu et al., 2021).

### 2.3 Important traits

#### 2.3.1 Single-cell C_4_ photosynthesis

*B. sinuspersici* is one of only four confirmed terrestrial plant species capable of performing C4 photosynthesis within a single chlorenchyma cell, without the Kranz anatomy that defines all other known C4 lineages (Sharpe and Offermann, 2014) In mature leaves, a large central vacuole partitions the chlorenchyma cell into two functionally distinct cytoplasmic domains: the peripheral compartment (PCC), which houses PEPC for initial CO2 fixation, and the central compartment (CCC), which houses Rubisco and NAD-malic enzyme (NAD-ME) for CO2 release and reassimilation via the Calvin cycle (Han et al., 2023) This intracellular carbon-concentrating mechanism suppresses photorespiration and achieves photosynthetic efficiency equivalent to classic two-cell C4 plants (Sharpe and Offermann, 2014). Young leaves initially display a uniform C3-type chloroplast distribution, transitioning to the dimorphic SCC4 arrangement as leaves mature a developmental plasticity that makes *B. sinuspersici* an ideal model for studying the molecular basis of C3–C4 photosynthetic transitions (Sharpe et al., 2025).

#### 2.3.2 Chloroplast compartmentalization and CHUP1-mediated organisation

The spatial segregation of PCC and CCC chloroplasts is physically maintained by CHUP1 (Chloroplast Unusual Positioning 1) and its associated actin-binding proteins (ABP) (Won et al., 2023) demonstrated that the CHUP1-like_a and CHUP1-like_b isoforms of *B. sinuspersici* are structurally enlarged relative to all C3, C4 (Kranz), and CAM species examined, and are absent in Arabidopsis establishing them as SCC4-specific structural proteins essential for the vacuole-mediated chloroplast partitioning that underpins single-cell C4 function. Co-expression of CHUP1 and ABP genes during leaf maturation further confirms their joint regulatory role in establishing intracellular compartmentation (Erlinghäuser, 2018).

#### 2.3.3 Altered energy metabolism: cyclic vs. linear electron flow

A key biochemical trait of *B. sinuspersici* is the spatial separation of cyclic electron flow (CEF) in PCC chloroplasts and linear electron flow (LEF) in CCC chloroplasts within a single cell (Han et al., 2023). identified, via comparative four-species transcriptomics, that CEF-associated genes are co-expressed with SCC4 cycle genes in mature leaves, whereas LEF genes are enriched in CCC. This dual electron-flow partitioning generates the ATP: NADPH ratio imbalance necessary to power the C4 pumping cycle, a metabolic innovation absent in Kranz C4 plants. The APE1 high-light acclimation protein and NITR2;1 chloroplastic nitrite transporter, both upregulated exclusively in SCC4 mature leaves but not in Kranz C4 species, further define the unique energetic signature of this trait (Lara et al., 2008).

#### 2.3.4 Leaf succulence and ion compartmentalization

The leaves of *B. sinuspersici* are semi-terete and succulent, with water-storage tissue (ws cells) adjacent to chlorenchyma, enabling ionic dilution under saline conditions (Yanagisawa and Chuong, 2022). Vacuolar Na+ sequestration via constitutively expressed NHX antiporters provides continuous ion protection prior to stress onset a pre-adaptive strategy distinct from the stress-inducible NHX expression seen in glycophytes. Simultaneously, differentiated antioxidant responses in PCC vs. CCC chloroplasts including compartment-specific iron superoxide dismutase (FeSOD) regulation protect the photosynthetic apparatus under both salinity and drought (Sharpe et al., 2025).

#### 2.3.5 Developmental plasticity and *in vitro* regenerability

*B. sinuspersici* supports direct *in vitro* shoot organogenesis from bud explants on Murashige and Skoog medium supplemented with 6-benzyladenine (BA), with highest regeneration at 4.4 μM BA after three weeks. Regenerated shoots retain the full intracellular SCC4 cytoplasmic compartmentalization on transplantation to soil (Northmore et al., 2012; Rosnow et al., 2014). This regeneration capacity is important for genetic transformation experiments aimed at functional gene validation.

### 2.4 Economic significance

#### 2.4.1 Genetic resource for crop salt tolerance

The primary economic value of *B. sinuspersici* lies in its utility as a genetic donor for salt tolerance improvement in staple crops. Salt stress already affects more than 6% of global land area and 20% of all irrigated land, directly threatening food security (Kidwai et al., 2021). Halophytes such as *B. sinuspersici* offer characterised stress-tolerance genes and alleles that are absent or functionally inferior in glycophytic crops. The transgenic transfer of BsHKT1;2 into Brassica rapa producing higher biomass and longer roots under salt stress relative to non-transgenic controls demonstrates a direct, validated pathway for exploiting *B. sinuspersici* genetic resources for commercial crop improvement (Irulappan et al., 2023). The species thus represents a living gene bank for novel ion transporter alleles, ERF transcription factors, and osmoprotectant pathway genes applicable to rice, wheat, and vegetable breeding programmes in salt-affected South Asian and Middle Eastern agricultural systems.

#### 2.4.2 Model system for C_4_ crop engineering

As one of only four known SCC4 species, *B. sinuspersici* carries outsized value as a biological template for the C4 rice engineering initiative a global effort estimated to have the potential to increase rice yield by 50% and reduce water consumption by 30–50% (Li et al., 2024). Unlike Kranz C4 donor plants (e.g., maize), *B. sinuspersici* provides a minimal-gene-set solution: its transit peptide (TP) sequences, which mediate selective protein targeting to PCC vs. CCC chloroplasts, have already been shown to be transferable to other species (Wimmer et al., 2017). The economic returns of successfully engineering even a partial SCC4 mechanism into C3 crops without requiring the structural redesign of Kranz anatomy would be transformative for global food production.

#### 2.4.3 Phytoremediation and saline soil rehabilitation

*B. sinuspersici* has potential as a phytoremediation agent for saline and sodic soils. Halophytes used in phytoremediation of salt-affected land extract Na+ and Cl− from soils, add organic matter, and restore essential cations (Ca2+, K+, N, P), improving long-term soil productivity (Aamer et al., 2024). Given *B. sinuspersici* ‘s constitutional tolerance of 50–200 mM NaCl the salinity range of most degraded agricultural soils it is well-suited for this application in the Persian Gulf region and broader South Asia, where approximately 34 million hectares are salt-affected in Iran alone (Aamer et al., 2024; Nainwal et al., 2024; Rosnow et al., 2014).

#### 2.4.4 Phytochemical and antimicrobial potential

Recent phytochemical characterisation of *B. sinuspersici* extracts reveals commercially relevant secondary metabolites, including total phenolics (34.2 mg/g), flavonoids (20.6 mg/g), tannins (17.6 mg/g), and gallic acid (2.22 mg/g) as the primary polyphenol identified by HPLC (Aamer et al., 2024). GC–MS analysis additionally identified oleic acid and n-hexadecanoic acid as the principal components. Antimicrobial assays using disk diffusion confirmed activity against phytopathogenic microorganisms, indicating potential for development of plant-derived biopesticides or antifungal agents for sustainable agriculture. These properties extend the economic significance of *B. sinuspersici* beyond genetics into the domains of nutraceuticals, natural product chemistry, and agrochemical development.

### 2.5 Domestication status

#### 2.5.1 Current domestication status

The published primary record still treats *Bienertia sinuspersici* as a wild model halophyte rather than a crop (Akhani et al., 2005). described it from natural southwest Asian populations as an annual species whose flowers are usually hermaphroditic and sometime unisexual, with flat seeds measuring about 1.87–2.30 × 1.67–2.03 mm. As summarized in the recent chromosome-level genome paper (Kim et al., 2025), the cultivation literature is experimental rather than agronomic: plants have been established from seed in common-garden and growth-chamber studies, grown hydroponically, and regenerated *in vitro*; seed propagation is feasible, but later propagation work reports limited seed stock together with low seed production and viability, which helps explain why the system shifted toward cuttings and tissue culture. Quantitative trait evidence is promising but still pre-crop. In hydroponics for ~7 weeks, 50–200 mM NaCl produced several-fold increases in root and shoot fresh and dry weight relative to 0 mM NaCl, maximum shoot dry weight occurred at 50 mM, leaf number increased about fourfold at 50 mM and about 3.4-fold at 100–200 mM, and mean individual leaf area rose from 0.36 cm² at 0 mM to 0.65 cm² at 200 mM; by contrast, 400 mM NaCl severely inhibited growth and reduced area-based Amax from 20.9 to 4.5 μmol CO_2_ m^−2^ s^−1^ (Kam et al., 2016). Drought and severe salinity also impose real penalties: after 10 days of water withholding, shoot length, shoot fresh weight, and root dry weight fell by 30%, 22%, and 32%, respectively, while 400 mM NaCl caused comparable declines; heat tolerance is well supported ecologically rather than experimentally, because the species grows in very hot coastal deserts and can reach 130 cm in height with canopies up to 160 cm under favourable conditions. Genetically, the new reference genome was assembled from a single-seed-derived line that still showed considerable genomic heterogeneity, indicating useful standing variation, but population-scale diversity panels, GWAS resources, seed-yield datasets, and crop-style multi-environment trials remain absent.

#### 2.5.2 Accelerated domestication potential

A realistic accelerated-domestication program should therefore start with broad wild germplasm capture followed by recurrent family selection for establishment, branching, flowering time, biomass, seed set, shattering/retention, and harvestability under moderate salinity and cyclic drought, rather than assuming a selfing-based ideotype on the outset. The major infrastructure barrier for genomics has now been removed (Kim et al., 2025) discovered a 3.608-Gb genome with 7,031 scaffolds, 9 chromosome-level scaffolds representing 89.5% of the genome, 40,465 annotated genes, and 95.7% BUSCO completeness, which is sufficient to support resequencing, pangenome construction, marker discovery, and once a phenotype diversity panel exists GWAS and genomic selection. Functional studies already point to tractable target modules for marker-assisted breeding or editing (Kumar et al., 2024). The salt-handling network is an obvious entry point because *B. sinuspersici* shows improved growth at 100 mM NaCl, BsHKT1;2 is strongly induced under moderate salt, and heterologous expression of 35S::BsHKT1;2 increased salt tolerance, biomass, canopy size, and root performance in *B. rapa* under salt stress (Irulappan et al., 2023); in parallel, transcriptomics implicates altered energy-metabolism programs in SCC4 establishment, and (Nguyen et al., 2025) identified two β-carbonic anhydrases, BsCAβ1 and BsCAβ2, that are strongly induced with leaf maturation and localize to the cytosol and plasma membrane, making them defensible mechanistic targets for improving carbon-concentrating efficiency under saline growth. Technically, the species is already unusually tractable for a wild halophyte: direct shoot induction from buds peaked at 4.4 μM BA, half-strength MS plus 1.0 mg L−1 IBA gave the best rooting and transplant survival from cuttings, 1.2% CO2 improved regeneration, and protoplast transfection exceeded 80%, so rapid clonal multiplication and early gene-function testing are realistic even though a stable heritable editing pipeline has not yet been published. Because BsHKT1;2 already works as a donor trait in *B. rapa*, whereas *B. sinuspersici* itself still lacks seed-yield, food/feed quality, and harvestability data, the most plausible near-term socioeconomic pathway is as a donor of stress-resilience/SCC4 modules and, secondarily, as a niche saline biomass crop rather than as an immediate grain crop; edited or transgenic lines would additionally face jurisdiction-specific oversight that is actively evolving, as recent revisions to biotechnology regulations in both the United States and the European Union illustrate (Canales & Fears, 2023; Hoffman, 2016).

## 3 Molecular phylogenetics and evolutionary history

The single-cell C4 photosynthesis of *Bienertia sinuspersici* is one of the most striking convergent innovations in vascular plant evolution and reconstructing its phylogenetic and evolutionary context is central to understanding both the origin of carbon-concentrating mechanisms in eudicots and the genetic resources this species offers for crop improvement. This section integrates evidence from nuclear ribosomal, plastid, and mitochondrial markers (Section 3.1), examines the divergence of Bienertia from its closest relatives Suaeda and Salicornia (Section 3.2), considers the palaeoclimatic forces that drove the adaptive radiation of single-cell C4 species in arid Southwest Asia (Section 3.3), and finally summarises what is known and what remains unknown about population genetics and phylogeography of the species (Section 3.4). A graphical timeline of these research milestones from 2002 to 2025 chromosome-level genome assembly is provided in **Supplementary Figure 2**.

### 3.1 Phylogenetic analyses (rDNA, cpDNA, mtDNA)

Phylogenetic placement of *B. sinuspersici* has been progressively refined through three categories of molecular markers: nuclear ribosomal DNA (rDNA, particularly ITS), chloroplast DNA (cpDNA), and chloroplast genome (plastome)-scale data (**Table 1**). Early multi-locus analyses combining nrITS, atpB-rbcL, and psbB-psbH sequences placed the genus Bienertia as a strongly supported monophyletic lineage within subfamily Suaedoideae, sister to Suaeda section Schoberia (Kapralov et al., 2006; Koteyeva et al., 2016). This placement was independently corroborated by chloroplast genome assemblies showing closest affinity to Salicornia species at the genome-wide level (Kim et al., 2016; Sharpe et al., 2020).

The first complete chloroplast genome of *B. sinuspersici* was assembled by (Kim et al., 2016) revealing a 153,472 bp circular plastome containing 127 genes (83 protein-coding, 36 tRNA, 8 rRNA) with a typical quadripartite structure. Maximum-likelihood phylogeny based on full plastome sequences from Chenopodiaceae recovered Bienertia as nested among Salicornia species, consistent with the broader Suaedoideae and Salicornioideae clade relationship documented by multi-locus studies. The most comprehensive plastome-scale analysis to date by (Liu et al., 2026), included 119 plastomes spanning all seven subfamilies of Chenopodiaceae and twelve independent C4 lineages. Their analysis identified five major IR/SC boundary types, with type V characterised by translocation of the trnH gene into both inverted-repeat regions occurring exclusively in two single-cell C4 Suaedoideae species, including *B. sinuspersici*, and absent from C3 relatives. This structural signature, combined with significant codon usage bias detected only in C4 species (Hagenau, 2017). Based on this (Liu et al., 2026), suggests plastome-level remodelling associated with the evolution of single-cell C4 photosynthesis.

Cytogenetic data complement molecular phylogenetic evidence (Sevilleno et al., 2020) used fluorescence *in situ* hybridisation (FISH) with 5S/45S rDNA and Arabidopsis-type telomeric probes to demonstrate that *B. sinuspersici* possesses 2n = 2x = 18 metacentric chromosomes (total length 83.07 μm), with 45S rDNA loci on chromosome 7 and 5S rDNA loci distributed across six chromosome pairs. A hemizygous 5S rDNA locus on chromosome 8 was interpreted as evidence of unequal crossing-over, a process consistent with ongoing molecular drive in the species’ rDNA arrays. The chromosome-level genome assembly of (Kim et al., 2025) subsequently confirmed nine chromosome-level scaffolds (3,608 Mbp; N50 = 360.8 Mbp; 40,465 annotated genes; BUSCO completeness > 95%), providing the genomic backbone for future high-resolution phylogenomic analyses.

In contrast to the well-resolved nuclear rDNA and plastid datasets, mitochondrial DNA remains a substantially under-utilised marker for *B. sinuspersici*. No mitochondrial genome assembly has been published for the species or, indeed, for any Bienertia taxon. This is a significant gap because plant mitochondrial genomes evolve at distinctive rates from plastid and nuclear genomes (Zwonitzer et al., 2024) and are highly informative for detecting cytonuclear discordance arising from ancient hybridization a process recently shown to be widespread across Amaranthaceae (Morales-Briones et al., 2021). Indeed (Liu et al., 2026), explicitly excluded ITS from divergence-time estimation owing to gene-tree discordance with plastid markers, citing (Morales-Briones et al., 2021) as evidence that ancient hybridization has produced reticulate phylogenetic patterns in this clade. Mitochondrial sequencing of Bienertia should therefore be a priority in future phylogenomic work.

Consistent with these published analyses, a maximum-likelihood phylogeny based on chloroplast rbcL sequences confirms placement of *B. sinuspersici* within the chenopod clade, sister to all other sampled Amaranthaceae taxa (**Supplementary Figure 1**; GenBank accession numbers in **Supplementary Table 1**).

**Table 1:** Molecular markers used in phylogenetic analyses of *B. sinuspersici* and their key contributions to placement, evolutionary inference, and identified gaps.

Marker/region	Genome/inheritance	Phylogenetic resolution	Key finding for *B. sinuspersici*
nrITS (ITS1–5.8S–ITS2)	Nuclear rDNA; biparental	Genus and species-level relationships within Suaedoideae; tests for hybridisation and discordance with plastid signal	Places Bienertia within Suaedoideae as sister to Suaeda; (Morales-Briones et al., 2021) report nuclear-plastid discordance suggesting ancient hybridisation
rbcL (cpDNA)	Plastid; uniparental (maternal)	Family- to subfamily-level placement; C_4_ evolution analyses across Amaranthaceae s.l.	Confirms placement in Suaedoideae and supports independent C_4_ origin distinct from Suaeda Borszczowia (Liu et al., 2026)
matK + atpB-rbcL (cpDNA)	Plastid; uniparental (maternal)	Hypervariable plastid markers identified by (Liu et al., 2026) for tribal-level resolution in Chenopodiaceae s.s.	Supports monophyly of tribe Bienertieae (Bienertia + Suaeda) as a Suaedoideae lineage
psbB-psbH + ITS combined	Plastid + nuclear	Resolves Suaedoideae internal relationships; differentiates Suaeda sections from Bienertia	Bienertia recovered as a strongly supported monophyletic genus sister to Suaeda sect. Schoberia (Kapralov et al., 2006)
Whole plastome (78 CDS)	Plastid; uniparental (maternal)	Genome-scale resolution; codon usage and structural-variant analyses	Bienertia plastome shows IR-boundary type V (trnH translocation) unique to SCC4 species; significant codon usage bias correlated with C_4_ evolution (Liu et al., 2026)
Mitochondrial DNA (mtDNA)	Mitochondrial; uniparental (maternal)	Slow-evolving in plants; useful for deep nodes and to detect cytonuclear discordance	Currently under-utilised in Bienertia; no published mitochondrial genome assembly to date a clear research gap

### 3.2 Divergence from Suaeda and Salicornia

Within Suaedoideae, Bienertia is consistently recovered as monophyletic and sister to Suaeda Forssk. ex J.F. Gmel., the largest and most ecologically diverse halophytic genus in Chenopodiaceae s.s. Multi-locus phylogenetic analyses combining nrITS, atpB-rbcL, and psbB-psbH place Bienertia closest to Suaeda section Schoberia, with strong bootstrap and Bayesian support (Kapralov et al., 2006). The monotypic genus Alexandra was reclassified within Suaeda as a result of the same analyses, leaving Bienertia as the only morphologically and physiologically distinct sister lineage to Suaeda within tribe Bienertieae (Brandt et al., 2015; Kapralov et al., 2006).

The relationship to Salicornia L. is more distant. Both Salicornia and Bienertia belong to the broader Salicornioideae – Suaedoideae clade, but they occupy separate subfamilies that diverged in the early Eocene, approximately 40 Ma based on plastome-calibrated divergence-time estimation (Liu et al., 2026; Steffen et al., 2015). The two subfamilies are sister groups (bootstrap = 100%, posterior probability = 1) and share a common ancestor that pre-dated the origin of single-cell C4 photosynthesis in either lineage confirming that the SCC4 mechanism in Bienertia arose independently of the evolution of two-cell C4 in Salicornioideae (Koteyeva et al., 2011; Liu et al., 2026).

(Liu et al., 2026; Piirainen et al., 2017) provides the most precise divergence-time estimates currently available. Suaedoideae diverged from Salicornioideae approximately 40.08 Ma (95% HPD: 52.12–28.54 Ma), with crown-group diversification of Suaedoideae beginning around 33.96 Ma (95% HPD: 50.08–14.89 Ma) at the Eocene–Oligocene transition. Critically, the Bienertia stem lineage was identified as the oldest C4 lineage within Chenopodiaceae s.s., dating to approximately 33.96 Ma. However, the crown age of Bienertia the divergence of the four extant species (B. cycloptera, *B. sinuspersici*, B. kavirense, and B. przewalskii) is much younger, estimated at only 1.60 Ma (95% HPD: 3.45–0.01 Ma) in the Pliocene–Pleistocene (Liu et al., 2026). The substantial gap (≈32 Myr) between the stem and crown of Bienertia suggests historical extinction of intermediate lineages, although the inferred Pliocene crown age may underestimate the true age if cryptic extinct lineages are not accounted for.

(Sharpe and Offermann, 2014) reviewed the broader evolutionary context of single-cell C4 photosynthesis and concluded that two independent origins are well supported within Suaedoideae: one in Bienertia and a separate origin in *Suaeda aralocaspica* (formerly Borszczowia aralocaspica). The two SCC4 lineages are estimated to have evolved between approximately 20.8–2.6 Ma for Bienertia and approximately 7.7 Ma to the present for S. aralocaspica, consistent with the Liu et al. (2026) estimate that the C4-Borszczowia lineage originated approximately 11.25 Ma (95% HPD: 19.7–4.85 Ma). Importantly, although both lineages share the same anatomical solutiondimorphic chloroplasts within a single chlorenchyma cell — they exhibit structurally distinct sub-cellular arrangements: Bienertia species have a central cytoplasmic compartment surrounded by a peripheral one, whereas S. aralocaspica partitions chloroplasts at opposite poles of elongated cells (Sharpe and Offermann, 2014). This anatomical divergence supports the hypothesis of independent evolutionary origins from a common Suaedoideae ancestor.

### 3.3 Palaeoclimatic influences and adaptive evolution

The origin of the Bienertia lineage at approximately 34 Ma coincides closely with the Eocene–Oligocene transition, a period of major global cooling, declining atmospheric CO2, and intensifying continental aridification (Lauretano et al., 2021). Using this (Liu et al., 2026) showed that this period also marks one of the two oldest C4 origins in Chenopodiaceae s.s. (the other being the Caroxyleae lineage in Salsoloideae at ≈32 Ma). The temporal coincidence of Bienertia stem origin with sustained atmospheric CO2 decline below 500 ppm (Hönisch et al., 2023) supports the long-standing hypothesis that CO2 starvation provided a key selective pressure for the evolution of carbon-concentrating mechanisms (Christin et al., 2008; Sage, 2004).

However, declining CO2 alone is insufficient to explain the spatial and temporal patterns of single-cell C4 diversification. (Rudov et al., 2020) demonstrated that Southwest Asia spanning the Persian Gulf basin, Iranian Plateau, and Arabian Peninsula hosts up to 20 of the 38 known independent eudicot C4 origins, including all known terrestrial single-cell C4 species. This regional diversity hotspot is attributable to a combination of factors: (i) high topographic and climatic heterogeneity; (ii) the presence of seven distinct phytogeographic zones, including the Irano-Turanian region as a centre of Caryophyllales diversification; and (iii) the prevalence of saline, hyper-arid lowland habitats that favour halophytic life-history strategies. C4 species richness in Chenopodiaceae increases with continentality and decreases with summer precipitation, identifying aridity and temperature extremes as the principal climatic drivers of the lineage’s distributional limits (Munroe et al., 2022).

A marked acceleration in the appearance of new C4 lineages within Chenopodiaceae s.s. occurred between approximately 20 and 15 Ma during the Mid-Miocene Climatic Optimum and subsequent cooling phase (Tauxe and Feakins, 2020). This Miocene radiation coincided with the broader expansion of C4 grasslands documented across continents, driven by the combined effects of sustained low atmospheric CO2, increased seasonal aridification, and the spread of fire-prone habitats. Although Bienertia did not undergo the explosive species-level radiation seen in C4 grass clades such as Chloridoideae, the genus persisted through this entire interval as a low-diversity lineage strictly confined to extremely saline desert habitats around the Persian Gulf and inland Iranian salt deserts (Akhani et al., 2005, 2012). **Table 2**. Chronological summary of key evolutionary research milestones on *Bienertia sinuspersici*, 1879–2025. Studies are listed by publication year and grouped by the type of evidence contributed (taxonomy, biochemistry, anatomy, molecular phylogeny, cell biology, genomics). Each entry indicates the principal finding and its evolutionary implication for understanding the origin of single-cell C_4_ photosynthesis.

**Table 2:** Key evolutionary, anatomical, physiological, and genomic studies in *Bienertia sinuspersici* research.

Key finding	Evidence	Evolutionary implication	References
Demonstrated C_4_ photosynthesis in *B. cycloptera* without Kranz anatomy, detailing its compartmentalization.	Biochemical and cell biology (microscopy, isotopic labelling)	Provided definitive proof that *Bienertia* performs C_4_ within single cells; established the cellular mechanism (dimorphic chloroplasts, central vs. peripheral compartments).	(Voznesenskaya et al., 2002)
Reviewed single-cell C_4_ in *Bienertia* and *Borszczowia*, noting that Kranz anatomy is not always required for C_4_ efficiency.	Conceptual synthesis (review)	Framed importance of *Bienertia* in C_4_ evolution; suggested engineering C_4_ in crops might not need Kranz anatomy.	(Sage, 2002, 2004)
Discovered and described *Bienertia sinuspersici* as a new species (second in genus) with single-cell C_4_ anatomy.	Morphology, anatomy, physiology, karyology	Showed distinct lineage separate from *B. cycloptera*, adapted to extreme heat; confirmed an additional independent origin of terrestrial SCC_4_.	(Akhani et al., 2005)
Used ITS and plastid sequences to clarify *Bienertia*’s phylogenetic position and C_4_ origins.	Molecular phylogeny (DNA sequence)	Confirmed *Bienertia* in Suaedoideae and identified it as one of two independent origins of non-Kranz C_4_ (parallel to *Suaeda* sect. *Borszczowia*); highlights convergent evolution of SCC_4_ in Chenopodiaceae.	(Kapralov et al., 2006)
Showed vacuole enlargement and cytoskeleton reorganization as crucial for forming two cytoplasmic domains in *B. sinuspersici* leaves.	Microscopy (light/electron), cell biology	Demonstrated that intracellular partitioning (analogous to Kranz BS/MC) is achieved via vacuolar and cytoskeletal dynamics; highlights cellular evolutionary innovation in SCC_4_.	(Park et al., 2009)
Mapped proteome and subcellular changes during development of SCC_4_ in *B. sinuspersici*.	Proteomics, cell biology	Identified proteins and structural changes key to forming two cytoplasmic domains, illuminating the ontogeny of SCC_4_ anatomy (evolutionary novelty).	(Offermann et al., 2015)
Described the structural and biochemical development of SCC_4_ along leaf gradients in *B. sinuspersici* and *S. aralocaspica*.	Anatomy, enzyme localization.	Showed how dimorphic chloroplasts and enzymes differentiate during leaf maturation; implies developmental parallels and constraints in independent SCC_4_ evolution.	(Koteyeva et al., 2016)
Analysed the YABBY transcription factor family in *B. sinuspersici*, revealing gene retention/loss patterns compared to C_3_/C_4_/CAM species.	Comparative genomics, phylogeny	Suggests regulatory gene evolution in *Bienertia* may underlie its leaf and cell polarity adaptations; hints at genetic shifts supporting SCC_4_.	(Soundararajan et al., 2019)
Used 3D electron tomography to examine thylakoid membrane assembly in *B. sinuspersici* chloroplasts.	Microscopy (electron tomography)	Found unique stacking patterns in central vs. peripheral chloroplasts; indicates structural adaptation of photosystems that evolved with SCC_4_.	(Mai et al., 2019)
Conducted cytogenetic (karyotype) analysis of *B. sinuspersici* as a step toward genome sequencing.	Cytogenetics (chromosome counts)	Established that *B. sinuspersici* has 2n=18 metacentric chromosomes; provides evolutionary context on genome structure and ploidy in SCC_4_ lineage.	(Sevilleno et al., 2020)
Identified shifts in energy metabolism (e.g. upregulation of ATP-related pathways) in mature *B. sinuspersici* leaves, via transcriptomics.	Transcriptomics (comparative gene expression)	Suggests that metabolic reprogramming (distinct from Kranz C_4_) underpins efficient SCC_4_ operation, reflecting evolutionary fine-tuning of energy use.	(Han et al., 2023)
Examined reactive oxygen species (ROS) regulation in the two chloroplast types during stress in *B. sinuspersici*.	Physiology (stress assays, ROS assays)	Found differential antioxidant responses in central vs. peripheral chloroplasts; implies evolutionary adaptation of redox control in SCC_4_ niche specialization.	(Uzilday et al., 2023)
Analysed the evolution of CHUP1 (chloroplast movement protein) and related genes across *B. sinuspersici* and other C_3_/C_4_/CAM plants.	Computational phylogenetics	Showed that *Bienertia* has distinct CHUP1 variants, reflecting evolutionary changes in chloroplast positioning mechanisms necessary for SCC_4_ compartmentation.	(Won et al., 2023)
The chromosome-level genome assembly of *Bienertia sinuspersici* produced a high-quality genome resource, including a 3.608 Gb assembly, 7,031 scaffolds, 40,465 annotated genes, and nine chromosome-level scaffolds	Genome assembly (PacBio, Hi-C), comparative genomics, gene annotation	This genome resource helps explain the genetic basis and evolutionary development of single-cell C4 photosynthesis in *B. sinuspersici*.	(Kim et al., 2025)

### 3.4 Population genetics and phylogeography

Compared to its phylogenetic and evolutionary context, the population genetics and phylogeography of *B. sinuspersici* remain surprisingly under-investigated. The species occurs across a geographically extensive but ecologically narrow range encompassing Iran, Iraq, Kuwait, Saudi Arabia, the United Arab Emirates, Qatar, Oman, and now southwestern Pakistan (GBIF.org, 2026), where populations are restricted to coastal sabkhas, salt marshes, and inland saline depressions around the Persian Gulf and northern Gulf of Oman. Despite this wide distribution, no published study has yet examined intraspecific genetic structure, geographic patterns of allele frequency, or signatures of historical demographic events such as range expansion or bottleneck information that is standard for most ecologically and economically significant halophytes.

Available genetic resources for Bienertia remain dominated by reference-genome and gene-family studies on a single accession or limited materials. The chromosome-level genome assembly of (Kim et al., 2025) was generated from a single seed-derived plant (designated R5-5) propagated by vegetative cuttings, and K-mer analysis of this single individual nevertheless revealed considerable genomic heterogeneity a hint that natural populations may harbour substantial standing genetic variation. Cytogenetic analysis confirms diploid status (2n = 2x = 18) but does not address allelic diversity within or among populations (Sevilleno et al., 2020).

Comparative biogeographic data are more informative for Bienertia at the genus level than at the species level. The four Bienertia species occupy spatially distinct climatic niches: *B. sinuspersici* occupies hot, low-elevation coastal lowlands of the Persian Gulf basin (Akhani et al., 2012); *B. cycloptera* occupies higher-elevation temperate and cold deserts of central and eastern Iran (Noori, 2024); B. kavirense is a narrow endemic restricted to a single 4–5 km strip on the margin of the Dasht-e Kavir salt desert in central Iran (Dehshiri, 2018); and B. przewalskii extends into Central Asia (Sanderson, 2017). This vicariant distribution suggests that climatic specialisation and habitat fragmentation rather than dispersal limitation alone has structured the genus, with each species adapted to a distinct combination of temperature, elevation, and salinity (Akhani et al., 2012; Rudov et al., 2020)

From a conservation genetics perspective, ongoing dam construction and accelerated desertification in Iran threaten the highly localised B. kavirense population, and broader habitat degradation across the Persian Gulf basin places *B. sinuspersici* populations at increasing anthropogenic risk (Chatziefthimiou et al., 2026). Population-level genetic surveys using SSR markers (already characterised by Liu et al., 2026, for related Chenopodiaceae s.s. species) or genotyping-by-sequencing approaches would help quantify standing genetic diversity, identify locally adapted populations, and prioritise conservation efforts. Such information is also essential for the strategic use of *B. sinuspersici* as a genetic donor in crop breeding programmes, where allelic diversity at stress-tolerance loci such as BsHKT1 may vary substantially across the species’ range.

## 4 Single-cell C_4_ photosynthesis: mechanisms and evolutionary origin

The C4 photosynthetic pathway evolved as a CO2-concentrating mechanism that suppresses the oxygenase activity of Rubisco and allows efficient carbon assimilation under conditions of high temperature, low atmospheric CO2, and water scarcity (Schlüter and Weber, 2020). In the vast majority of C4 plants, this concentrating mechanism is supported by Kranz anatomy a specialized two-cell arrangement in which mesophyll cells (MC) initially fix CO2 via PEPC and bundle-sheath cells (BSC) decarboxylate the resulting C4 acids to release CO2 near Rubisco. *Bienertia sinuspersici* is one of only four terrestrial species known to operate a fully functional C4 cycle within an individual chlorenchyma cell, without Kranz anatomy (Edwards et al., 2004; Voznesenskaya et al., 2002). This section examines the structural and biochemical basis of single-cell C4 (SCC4) photosynthesis, the ion-regulatory machinery that supports it under saline conditions, and the evolutionary forces that drove its origin in the Suaedoideae.

### 4.1 Single-cell vs two-cell C_4_: key differences

In Kranz C4 plants, photosynthetic functions are spatially partitioned across two morphologically and biochemically distinct cell types arranged in concentric rings around the vascular bundles (Bräutigam and Gowik, 2016). Atmospheric CO2 enters the mesophyll, is hydrated to bicarbonate by carbonic anhydrase, and is fixed by phosphoenolpyruvate carboxylase (PEPC) to form oxaloacetate, which is converted to malate or aspartate. These C4 acids diffuse through plasmodesmata into the bundle-sheath cells, where they are decarboxylated by NADP-malic enzyme, NAD-malic enzyme, or PEP carboxykinase to release CO2 near Rubisco (Schlüter and Weber, 2020). The reinforced bundle-sheath cell wall and high plasmodesmatal density act as a diffusion barrier that maintains an elevated CO2 partial pressure around Rubisco.

**Figure 2:**
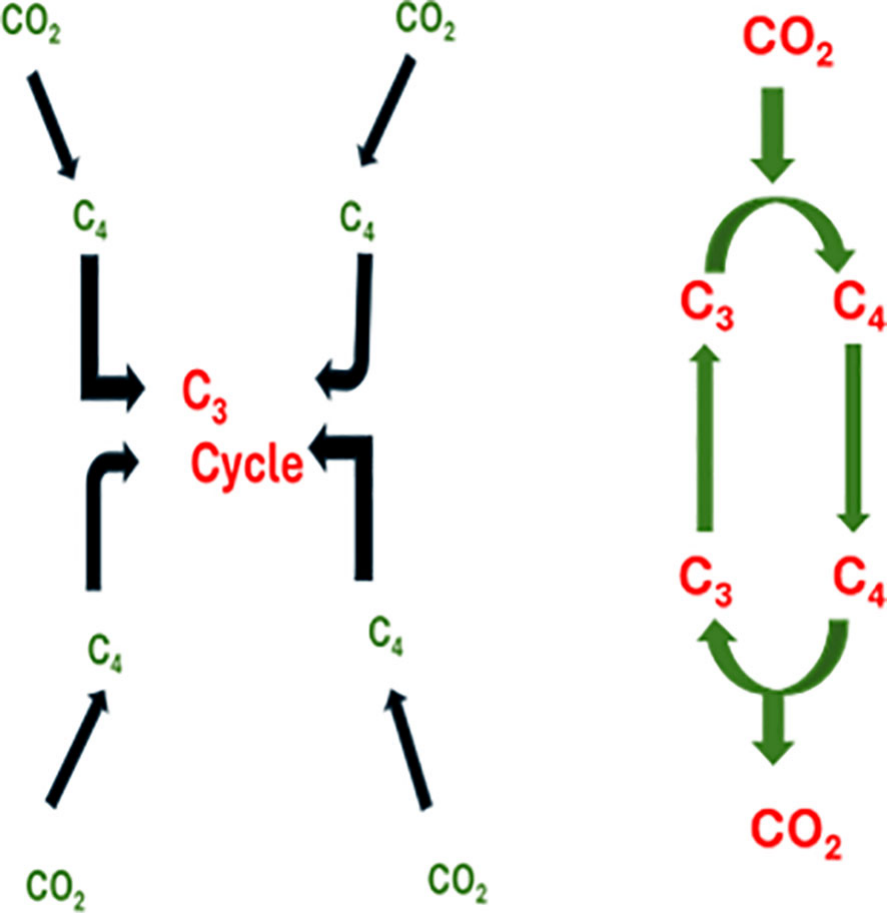
A two-panel figure illustrating (left) how C_4_ metabolism can be arranged inside a single photosynthetic cell (Chuong et al., 2006). and a conceptual depiction of the (right) C_3_ ↔ C_4_ metabolic cycle (Edwards et al., 2004).

In contrast, *B. sinuspersici* achieves the same biochemical outcome within a single chlorenchyma cell. The cell contains two structurally and biochemically dimorphic populations of chloroplasts peripheral chloroplasts (PCs) located near the plasma membrane and central chloroplasts (CCs) clustered around the nucleus in a central cytoplasmic compartment, separated by a large central vacuole transected by cytoplasmic channels (Edwards et al., 2004; Voznesenskaya et al., 2002). PEPC, pyruvate orthophosphate dikinase (PPDK), and the C4 cycle enzymes are localised to the peripheral compartment, whereas Rubisco, NAD-ME, and the Calvin cycle enzymes are restricted to the central compartment. The diffusion barrier provided by the cell wall and bundle-sheath geometry in Kranz C4 is replaced in SCC4 by the central vacuole and the long cytoplasmic distance between the two compartments (Edwards et al., 2004). Despite the absence of Kranz anatomy, the photosynthetic efficiency and CO2 compensation point of *B. sinuspersici* are comparable to those of two-cell C4 plants, demonstrating that intracellular compartmentalization is sufficient to operate a complete C4 cycle (Schlüter and Weber, 2020). **Figure 2** illustrates the C4 metabolic cycle and its organization within a single photosynthetic cell.

### 4.2 Intracellular compartmentalization in *B. sinuspersici*

**Figure 3:**
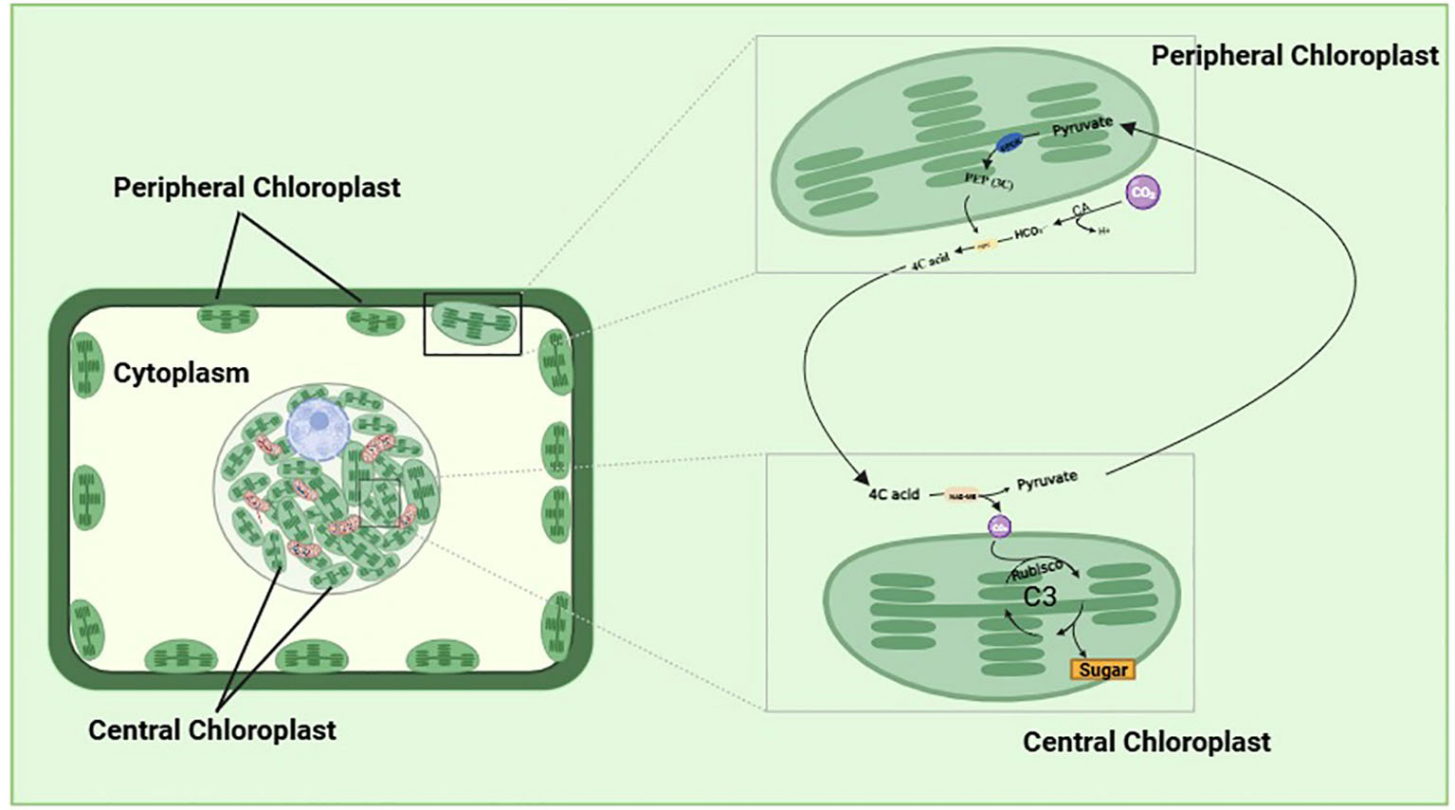
Schematic representation showing intracellular compartmentalization of photosynthetic functions within a single chlorenchyma cell. The peripheral chloroplasts (PC) near the cell wall fix bicarbonate (HCO_3_^-^) into four-carbon acids via phosphoenolpyruvate carboxylase (PEPC). These C_4_ acids are transported to the central compartment containing the central chloroplasts (CC) and mitochondria (M). In the CC, NAD-malic enzyme (NAD-ME) decarboxylates the C_4_ acids, releasing CO_2_ that is refixed by ribulose-1,5-bisphosphate carboxylase/oxygenase (Rubisco) through the Calvin (C_3_) cycle, producing sugars. Pyruvate, a by-product of this reaction, is transported back to the PC where pyruvate, phosphate dikinase (PPDK) regenerates phosphoenolpyruvate (PEP), completing the C_4_ cycle. This spatial separation of C_4_ and C_3_ reactions within a single cell allows *B. sinuspersici* to concentrate CO_2_ around Rubisco, enhancing photosynthetic efficiency and reducing photorespiration without Kranz anatomy.

The defining cytological feature of *B. sinuspersici* is the partitioning of a single chlorenchyma cell into two functionally distinct cytoplasmic domains (**Figure 3**). In mature leaves, an enlarged central vacuole pushes the cytoplasm into a thin peripheral layer containing the PC chloroplasts, while a dense central cytoplasmic compartment containing the CC chloroplasts, mitochondria, and the nucleus is suspended within the vacuolar lumen and connected to the periphery by cytoplasmic strands (Park et al., 2009). The actin and microtubule cytoskeletons play a critical role in establishing and maintaining this partition: pharmacological disruption of either cytoskeletal system in mature cells causes dispersion of the central compartment and loss of organelle organisation, abolishing functional SCC4 capacity (Chuong et al., 2006; Park et al., 2009).

Subcellular fractionation and immunolocalisation studies have confirmed the differential biochemical specialisation of the two chloroplast populations (Offermann et al., 2011). developed a density-gradient method to separate PC and CC chloroplasts and demonstrated by Western blotting that PEPC, PPDK, and pyruvate transporters are restricted to PCs, whereas Rubisco large and small subunits, NAD-ME, and Calvin cycle enzymes are confined to CCs. Mitochondria, which contain NAD-ME activity essential for C4 acid decarboxylation in NAD-ME type C4 plants, also localise specifically to the central compartment, confirming the operation of an NAD-ME-type SCC4 cycle.

Three-dimensional electron tomography by (Mai et al., 2019) revealed that the dimorphic chloroplasts also differ in thylakoid architecture: PCs contain reduced grana stacking and an expanded stroma thylakoid network, whereas CCs have well-developed grana similar to those of C3 mesophyll chloroplasts. This structural divergence supports differential allocation of photosystem I and photosystem II activity between the two compartments and parallels the granal differentiation observed between mesophyll and bundle-sheath chloroplasts of NAD-ME Kranz species. The compartmentalised architecture is established progressively during leaf maturation: young leaves contain a single uniform population of C3-like chloroplasts, and the dimorphic SCC4 arrangement emerges as cells expand and the central vacuole forms (Soundararajan et al., 2019). Differential expression of the YABBY family of polarity-determining transcription factors during this transition has been implicated in establishing the spatial cues that guide chloroplast and organelle positioning (Soundararajan et al., 2019). Functional validation of these regulatory networks has been enabled by the development of protoplast isolation and transient gene-expression protocols specific to *B. sinuspersici* (Lung et al., 2011), making this species an increasingly tractable system for cell-biological investigation of single-cell C4 photosynthesis.

### 4.3 Ion regulation and osmoregulation supporting C_4_ in saline conditions

**Figure 4:**
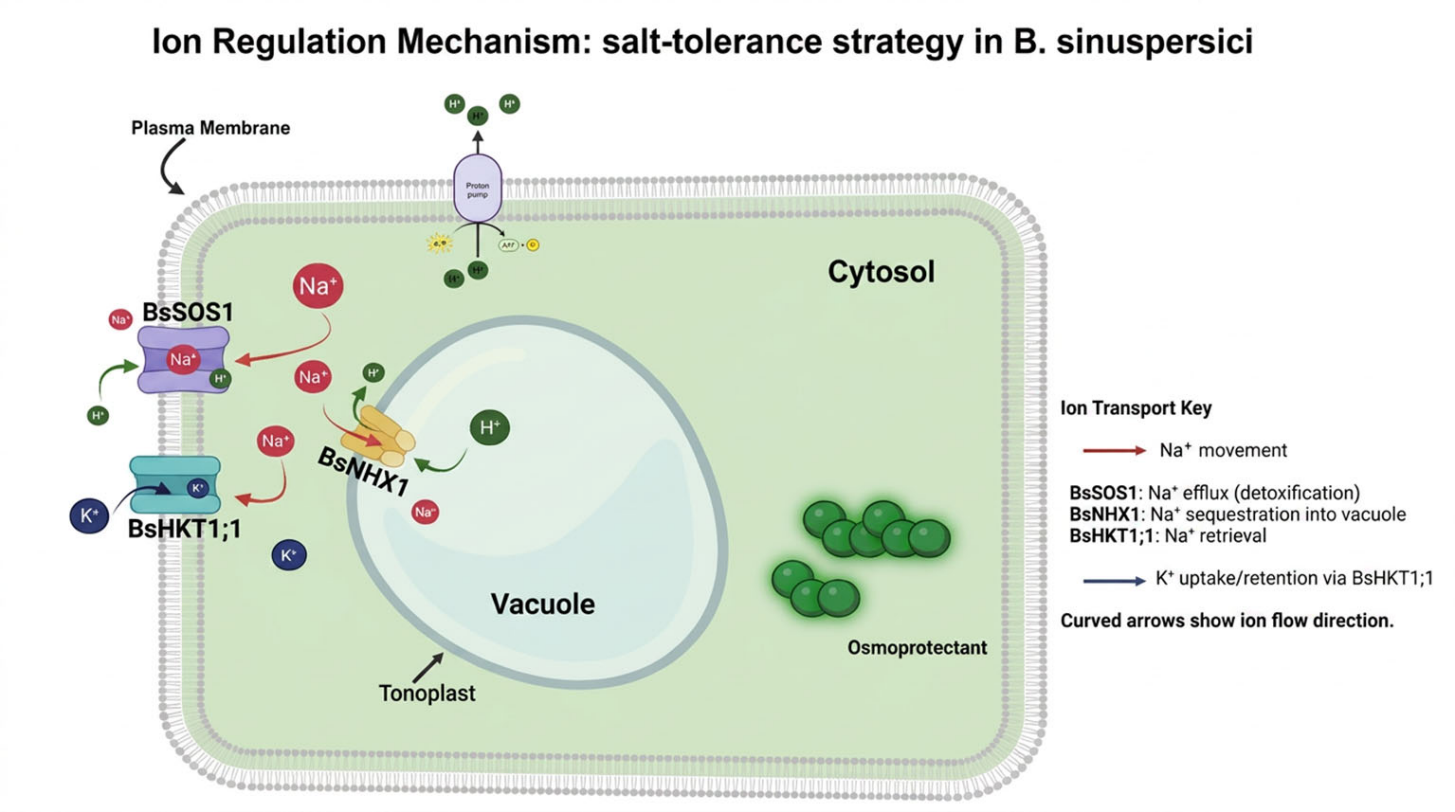
Schematic diagram shows how *B. sinuspersici* regulates ion balance under saline conditions. The plasma-membrane Na^+^/H^+^ antiporter BsSOS1 extrudes Na^+^ from the cytosol into the apoplast, energized by the proton gradient generated by H^+^-ATPase proton pumps. The tonoplast-localised BsNHX1 sequesters cytosolic Na^+^ into the vacuole, simultaneously contributing to osmotic adjustment. BsHKT1;1 mediates Na^+^ retrieval from the xylem and selective K^+^ retention in shoot tissues, preserving a favourable cytosolic K^+^/Na^+^ ratio. Compatible solutes (Osmo protectants) accumulate in the cytosol to balance vacuolar osmotic pressure.

Because *B. sinuspersici* naturally inhabits coastal sabkhas and inland saline depressions, the operation of its SCC4 cycle is inseparable from constitutive salt-tolerance machinery. Maintenance of a low cytosolic Na+ concentration and a high cytosolic K+/Na+ ratio is essential for the activity of carbon-fixation enzymes PEPC and Rubisco are both inhibited by elevated cytosolic Na+ making ion homeostasis a prerequisite for SCC4 function under field conditions (Van Zelm et al., 2020; Yang and Guo, 2018). Three transport systems operate in concert to achieve this homeostasis in *B. sinuspersici*: the SOS pathway, the NHX-type vacuolar antiporters, and the HKT-family high-affinity K+/Na+ transporters (Ma et al., 2024; Mansour and Hassan, 2022). **Figure 4** show ion-regulation strategy of *B. sinuspersici*.

### 4.4 Evolutionary drivers of the single-cell system

The repeated evolution of C4 photosynthesis at least 62 independent origins across vascular plants, including 22 in eudicots is one of the most striking examples of convergent biochemical evolution in nature (Sage, 2017). Within Chenopodiaceae, C4 photosynthesis arose at least twelve times, and within Suaedoideae alone there are at least four independent origins, including the SCC4 lineages of Bienertia and *S. aralocaspica* (Stata et al., 2019). The convergence of two anatomically and developmentally distinct SCC4 solutions within a single subfamily indicates that Suaedoideae shares ancestral pre-conditions succulent leaf anatomy, halophytic habitat, large central vacuoles, and a flexible chloroplast-positioning system that lowered the evolutionary barrier to single-cell carbon-concentrating mechanisms (Lundgren, 2020).

Three coupled selective pressures appear to have driven the origin of SCC4 in *Bienertia*. First, atmospheric CO2 decline through the Eocene–Oligocene transition (~34 Ma) reduced the operating efficiency of Rubisco and increased the relative cost of photorespiration, providing strong selection for any mechanism that could concentrate CO2 around Rubisco (Bräutigam and Gowik, 2016; Sage, 2017). Second, the progressive aridification and warming of Southwest Asia from the late Eocene onwards intensified water deficit and thermal stress in lowland habitats (Schlüter and Weber, 2020). Third, the colonisation of saline deserts imposed concurrent ionic stress, favouring lineages that combined CO2 concentration with salt-tolerance machinery a combination *Bienertia* achieves through the integration of compartmentalised C4 biochemistry with constitutive SOS, NHX, and HKT transporter activity (Mansour and Hassan, 2022; Stata et al., 2019). The vicariant split between *B. sinuspersici*, restricted to hot, saline Gulf habitats, and *B. cycloptera*, occupying cooler temperate settings, is consistent with stronger heat- and salinity-driven selection in the Gulf lineage (Akhani et al., 2005), and the unusually long stem branch leading to *Bienertia* (~32 Myr) suggests the SCC4 lineage persisted through the Miocene as a low-diversity halophytic specialist (Lundgren, 2020).

Comparative genomic and transcriptomic analyses indicate that the transition to SCC4 did not require qualitatively new genes; rather, it relied on quantitative changes in expression, alternative splicing, and the acquisition of novel transit peptides that direct chloroplast-targeted proteins to one of two organelle populations within the same cell (Schlüter and Weber, 2020). Biophysical modelling supports a stepwise origin, showing that in a *Bienertia*-like geometry only ~10 µm separation between primary and secondary carboxylases may be sufficient for SCC4 benefit, so cell enlargement, central mitochondrial localisation, and vacuolar partitioning could all have acted as pre-adaptive intermediates (Jurić et al., 2017). Recent genome-scale work chromosome-level assembly, developmental transcriptomics, YABBY and CHUP1 analyses, and salt-transporter expansion further indicates that SCC4 arose through coordinated changes in developmental timing, protein targeting, chloroplast positioning, and ion ecology rather than a single master switch (Han et al., 2023; Kim et al., 2025; Soundararajan et al., 2019; Won et al., 2023). This evolution-by-redeployment is consistent with the broader conclusion that C4 syndromes arise through co-option of pre-existing C3 regulatory networks rather than the invention of new ones (Sage, 2017; Stata et al., 2019). The success of *Bienertia* in the hyper-arid, hyper-saline Persian Gulf basin demonstrates that SCC4 is an evolutionarily viable solution, and its molecular components particularly the spatial-targeting transit peptides and integrated ion-transport machinery represent valuable genetic resources for engineering similar carbon-concentrating capability into staple C3 crops (Lundgren, 2020).

## 5 Genomic, transcriptomic, and genetic studies related to C_4_ pathway and stress tolerance

This section consolidates molecular evidence relevant to the C4 pathway and abiotic stress tolerance of Bienertia sinuspersici, drawing on genomic, transcriptomic, and where available mapping data. Three datasets currently dominate this evidence base: the chromosome-level reference genome assembly (Kim et al., 2025), the comparative transcriptome studies of C4 energy metabolism and developmental gradients (Han et al., 2023; Sharpe et al., 2025), and the targeted gene-family analyses of stress-tolerance and chloroplast-positioning loci (Ma et al., 2024; Won et al., 2023). Critically, no QTL maps or genome-wide association studies have yet been published for *B. sinuspersici* itself a gap that significantly limits direct allele discovery and is discussed explicitly in Sections 5.2 and 5.3. Section 5.6 synthesises the breeder-relevant alleles identified across all available studies into a priority table for crop-improvement programmes.

### 5.1 Genome organization and gene family expansion

The chromosome-level reference genome of *B. sinuspersici* using a combination of PacBio HiFi long-read sequencing, 10X Genomics linked reads, and Hi-C scaffolding. The final assembly is 3,608 Mbp distributed across 7,031 scaffolds, with nine chromosome-level scaffolds exceeding 100 Mbp that together cover 89.5% of the genome and correspond to the nine pairs of metacentric chromosomes (2n = 2x = 18) confirmed by FISH karyotyping (Sevilleno et al., 2020). The contig N50 is 360.8 Mbp, BUSCO completeness exceeds 95% on the embryophyta lineage dataset, and 40,465 protein-coding genes were annotated using IsoSeq full-length transcripts together with comparative protein evidence from Amaranthus hypochondriacus, *B. vulgaris*, *C. quinoa*, and *Suaeda aralocaspica* (Kim et al., 2025).

A striking feature of the genome is its high repetitive-element content: 79.02% of the assembly consists of transposable elements and other repeats, dominated by long terminal repeat retrotransposons (LTRs, 44.81%) followed by long interspersed nuclear elements (8.57%) and DNA transposons (7.77%). This repetitive load is comparable to that of other large-genome halophytes and is consistent with the species’ diploid 2n = 18 karyotype rather than recent polyploidy (Kim et al., 2025). The plastome is much more compact at 153,472 bp with the canonical quadripartite structure and 127 genes, but exhibits a unique IR/SC boundary configuration (type V) characterised by translocation of the trnH gene into both inverted-repeat regions a structural signature shared only with other single-cell C4 Suaedoideae and absent from C3 relatives (Liu et al., 2026).

Gene-family expansions of direct relevance to C4 function and salt tolerance have been documented from this assembly. The HKT1 high-affinity K+/Na+ transporter family is expanded into three paralogs (BsHKT1;1, BsHKT1;2, BsHKT1;3), in contrast to the single HKT1 copy in Arabidopsis (Ma et al., 2024). The CHUP1 (Chloroplast Unusual Positioning 1) gene, essential for vacuole-mediated chloroplast partitioning in SCC4 cells, occurs as two structurally enlarged isoforms, BsCHUP1-like_a and BsCHUP1-like_b, that are absent from Arabidopsis, Kranz C4 species, and CAM model plants making them a strong SCC4-specific gene-family expansion (Won et al., 2023). Other expansions of breeder interest include the NHX-type vacuolar antiporters, the ERF transcription-factor family (with one halophyte-type member, BsSERF-1, particularly upregulated under stress), and the YABBY family of leaf-polarity transcription factors (Soundararajan et al., 2019). The integration of these expansion patterns with the chromosome-level assembly creates a foundation for synteny-based gene discovery in related Chenopodiaceae crops.

### 5.2 QTL mapping for salt and drought tolerance

No published quantitative trait locus (QTL) studies are currently available for *B. sinuspersici*. This reflects the species’ status as a non-domesticated wild halophyte: there are no established mapping populations, no genetic recombinant inbred lines (RILs), and no segregating F2 populations the standard prerequisites for forward-genetic QTL mapping (Roy et al., 2014). Cytogenetic confirmation of diploid status (2n = 18; Sevilleno et al., 2020) and the chromosome-level reference assembly now make such crosses technically possible, but no breeding programme has been initiated.

In the absence of direct QTL data, comparative QTL studies in closely related Amaranthaceae crops provide the most informative analogues. Quinoa (*C. quinoa*) QTL mapping has identified salt-tolerance loci linked to homologues of SOS1, NHX1, and HKT1 with effects on shoot Na+ exclusion and biomass under saline conditions (Maughan et al., 2019; Patiranage et al., 2022). Sugar beet (Beta vulgaris), the chenopod genome most closely related to Bienertia at the plastome level (Park, 2018), has yielded salt-tolerance QTLs on chromosomes 4 and 9 that co-locate with ion-homeostasis genes (Skorupa et al., 2019). Synteny-based projection of these QTL intervals onto the *B. sinuspersici* assembly is now feasible and represents a high-value strategy for prioritising candidate alleles before mapping populations are developed in the species itself.

Establishing a Bienertia mapping population would require either intraspecific crosses among phenotypically diverse wild accessions across the Persian Gulf basin (genetic diversity which has not yet been characterised, see Section 3.4) or interspecific crosses with *B. cycloptera* or B. kavirense, which occupy contrasting climatic envelopes. Given the species’ demonstrated regenerability *in vitro* and amenability to vegetative propagation (Kim et al., 2025; Northmore, 2010; Rosnow et al., 2014) generation of segregating populations from such crosses is technically tractable and should be prioritized as a community resource.

### 5.3 GWAS and SNP discovery

No genome-wide association studies have been reported for *B. sinuspersici*. Genome-wide association studies in related species show rich natural variation that could be tapped for Bienertia improvement. In quinoa, re-sequenced 310 accessions (~2.9M SNPs) and found ~600 significant SNPs for 17 agronomic traits, including seed weight and disease resistance (Patiranage et al., 2022). Although salt tolerance was not directly mapped in that GWAS, it established the framework for SNP-based trait mapping in Chenopodiaceae. In sugar beet, performed GWAS for seedling-stage drought tolerance and identified 11 significant loci (SNPs on Chr2,3,5,7,9) linked to traits like root/leaf biomass; candidate genes cluster in sugar metabolism, osmoprotection and stress‐repair pathways (Li et al., 2023). Bienertia gains an edge from its new genomic resources: the chromosome-scale genome (3.61 Gbp, 40,465 genes) provides a dense marker map and pangenome potential (Kim et al., 2025). Moreover, Bienertia harbours unusually robust salt‐tolerance genes: BsHKT1;2 is highly induced by moderate salinity and confers Na^+ exclusion (transgenics overexpressing BsHKT1;2 showed greater biomass under salt), and two β-CA isoforms (BsCAβ1, BsCAβ2) are strongly upregulated during leaf maturation, with BsCAβ2 targeted to the plasma membrane for CO_2_ hydration (Nguyen et al., 2025). These unique alleles (inducible transporters and CA genes) coupled with genomic tools position *B. sinuspersici* to surpass its crop relatives in breeding for salt/drought resilience.

To progress toward true GWAS-driven allele discovery, a coordinated effort is needed to (i) sequence multiple wild accessions of *B. sinuspersici* sampled across its biogeographic range (Iran, Iraq, UAE, Oman, Pakistan, Saudi Arabia), (ii) phenotype these accessions for salinity, drought, and heat tolerance under controlled conditions, and (iii) develop a population-scale SNP genotyping assay anchored to the Kim et al. (2025) reference. Such resources would unlock direct allele-trait associations of high relevance to crop breeding (Negrão et al., 2017).

### 5.4 Transcriptome under salt and drought stress

Transcriptomic data unlike QTL or GWAS are comparatively well developed for *B. sinuspersici*. Three complementary studies provide the current evidence base. Comparative transcriptomes from *B. sinuspersici* and three reference C3/Kranz C4 species and identified an SCC4-specific reprogramming of energy metabolism. Cyclic electron flow (CEF)-associated genes are co-expressed with C4 cycle genes in mature leaves and localise to the peripheral compartment, while linear electron flow (LEF)-associated genes are enriched in the central compartment producing the ATP: NADPH stoichiometry required to support the C4 pumping cycle. The APE1 high-light acclimation protein and the NITR2;1 chloroplastic nitrite transporter were both upregulated exclusively in mature SCC4 leaves and absent from Kranz C4 controls (Han et al., 2023).

(Sharpe et al., 2025) extended this analysis with a developmental transcriptome contrasting young (1–3 mm) and mature (>1 cm) *B. sinuspersici* leaves. Young leaves operate predominantly C3-like photosynthesis with monomorphic chloroplasts and high photorespiratory enzyme expression, whereas mature leaves activate the full SCC4 gene set including PEPC, PPDK, NAD-ME, and compartment-specific FeSOD isoforms. Differential FeSOD regulation between PCC and CCC chloroplasts represents a previously unrecognised antioxidant strategy that protects the photosynthetic apparatus under combined drought and salinity. Complemented these findings with explicit drought- and salt-stress treatments, demonstrating that reactive oxygen species (ROS) regulation differs between the two chloroplast populations a compartmentalised redox response that mirrors the spatial separation of the C4 cycle itself (Uzilday et al., 2023).

Across these datasets, salt- and drought-responsive differentially expressed genes (DEGs) consistently include the BsSOS1, BsNHX1, and BsHKT1 paralogs, the osmolyte biosynthetic enzymes BsP5CS (proline) and BsBADH (glycine betaine), the BsSERF-1 ERF transcription factor, and chaperone networks (HSPs, dehydrins). These DEGs converge on the same molecular pathways characterised in halophytic transcriptomes more broadly (Ma et al., 2024; Zhang et al., 2020) and identify a coherent set of candidate genes for downstream functional validation in crops.

### 5.5 Novel alleles and uncharacterized pathways

**Table 3** synthesises twelve breeder-priority alleles and gene families identified from the genomic, transcriptomic, and functional studies reviewed above. Entries are organised by molecular pathway and indicate (i) the specific novelty of the *B. sinuspersici* allele relative to glycophytic crop counterparts, and (ii) the most promising breeding target for translational deployment. Alleles in the salt-tolerance pathway (BsSOS1, BsNHX1, BsHKT1 paralogs) are the most validated and translation-ready, with (Irulappan et al., 2023) already demonstrating direct transgenic transfer of BsHKT1;2 to Brassica rapa. The C4 cycle alleles and SCC4-specific components (BsCHUP1, transit peptides, BsAPE1, BsNITR2;1) represent a longer-term but transformative opportunity for the C4 rice initiative and analogous engineering of partial C4 cycles in C3 cereals. The full comparative gene family analysis is provided in **Supplementary Table 2**.

**Table 3:** Breeder-priority novel alleles and uncharacterized pathways from *B. sinuspersici*.

Gene/allele	Pathway	Novelty in *B. sinuspersici*	Breeding target	Reference
BsHKT1;1, BsHKT1;2, BsHKT1;3	Na^+^/K^+^ transport	Three-paralog expansion of HKT1; absent in Arabidopsis and rice; BsHKT1;2 transgenic B. rapa shows higher biomass and longer roots under salt stress	Rice, wheat, Brassica salinity tolerance via direct transgene transfer or marker-assisted introgression	(Irulappan et al., 2023)
BsSOS1	Plasma-membrane Na^+^ efflux	Constitutively expressed at high levels relative to glycophytes; structural features characteristic of fully active halophyte SOS1	Reduce shoot Na^+^ loading in saline-irrigated cereals; combine with SOS3–SOS2 stack	(Ali et al., 2021; Irulappan et al., 2023)
BsNHX1	Vacuolar Na^+^ sequestration	Constitutively (not stress-induced) expressed; supports succulence and osmotic adjustment at 50–200 mM NaCl optimum	Pre-emptive vacuolar storage for vegetable crops; durable salinity tolerance in spinach and quinoa	(Park et al., 2009; Urbanavičiūtė et al., 2021b)
BsCHUP1-like_a, BsCHUP1-like_b	Chloroplast positioning (SCC_4_-specific)	Enlarged CHUP1 isoforms unique to SCC_4_; absent in C_3_, Kranz C_4_, and CAM species; essential for vacuole-mediated chloroplast partitioning	Building block for engineering single-cell C_4_ mechanisms into C_3_ crops (rice C_4_ project)	(Park et al., 2009; Won et al., 2023)
BsPEPC	C_4_ cycle entry (carboxylation)	Halophyte-type C_4_ PEPC; reduced malate inhibition under saline conditions; optimised for cytoplasmic operation in single-cell context	Improve initial CO_2_ capture efficiency in C_3_ crops; salt-stable variant for engineered C_4_ rice	(Offermann et al., 2015; Wimmer et al., 2017)
BsPPDK	PEP regeneration	High specific activity in peripheral chloroplasts; efficient PEP regeneration coupled to BASS2 pyruvate transport	Sustain PEP supply in transgenic C_3_ crops attempting partial C_4_ cycle operation	(Han et al., 2023; Lara et al., 2008; Offermann et al., 2015)
BsNAD-ME	Mitochondrial C_4_ acid decarboxylation	Mitochondria localised exclusively to central compartment; tight coupling between decarboxylation and Rubisco fixation	Engineering NAD-ME-type C_4_ cycles into mitochondria-rich crop tissues	(Chuong et al., 2006; Han et al., 2023; Park et al., 2009)
Transit peptides (TP) for PCC vs CCC targeting	Selective protein localisation	Sequence elements direct nuclear-encoded proteins to peripheral or central chloroplasts within the same cell; transferable to other species	Synthetic biology toolkit for compartment-specific protein targeting in engineered plants	(Offermann et al., 2015; Wimmer et al., 2017)
BsAPE1, BsNITR2;1	Chloroplast energy partitioning	Upregulated exclusively in mature SCC_4_ leaves; absent or low in Kranz C_4_; supports CEF/LEF spatial separation	Maintain ATP: NADPH balance in C_4_-engineered C_3_ crops; high-light tolerance	(Han et al., 2023; Maeda et al., 2014; Walters et al., 2003)
BsSERF-1	Stress-responsive ERF transcription factor	Halophyte-type ERF; absent or weakly expressed in mainstream cereal and Brassica varieties; salt-induced upregulation	Master-regulator transgene for multi-stress tolerance (salinity + drought) in rice and wheat	(Mehra et al., 2024; Wani et al., 2020)
BsP5CS, BsBADH	Osmolyte biosynthesis (proline, glycine betaine)	Constitutive cytosolic osmolyte accumulation balancing vacuolar Na^+^; documented at ~2,000 mmol kg^-^¹ solvent under saline conditions	Stack with BsNHX1 for combined ionic and osmotic tolerance; rice and tomato applications	(Mehra et al., 2024; Ozturk et al., 2021; Urbanavičiūtė et al., 2021b)
BsFeSOD (compartment-specific)	Antioxidant defence (ROS scavenging)	Differential FeSOD regulation between PCC and CCC chloroplasts; protects photosynthetic apparatus under combined drought and salt stress	Targeted ROS protection in chloroplasts of stress-prone crops; pair with chloroplast transformation	(Uzilday et al., 2023)

Entries are based on the chromosome-level genome assembly (Kim et al., 2025), targeted gene-family analyses (Won et al., 2023), comparative transcriptomes (Han et al., 2023; Sharpe et al., 2025), and validated transgenic studies (Wimmer et al., 2017; Irulappan et al., 2023).

## 6 Applications for crop improvement in South Asia

**Figure 5:**
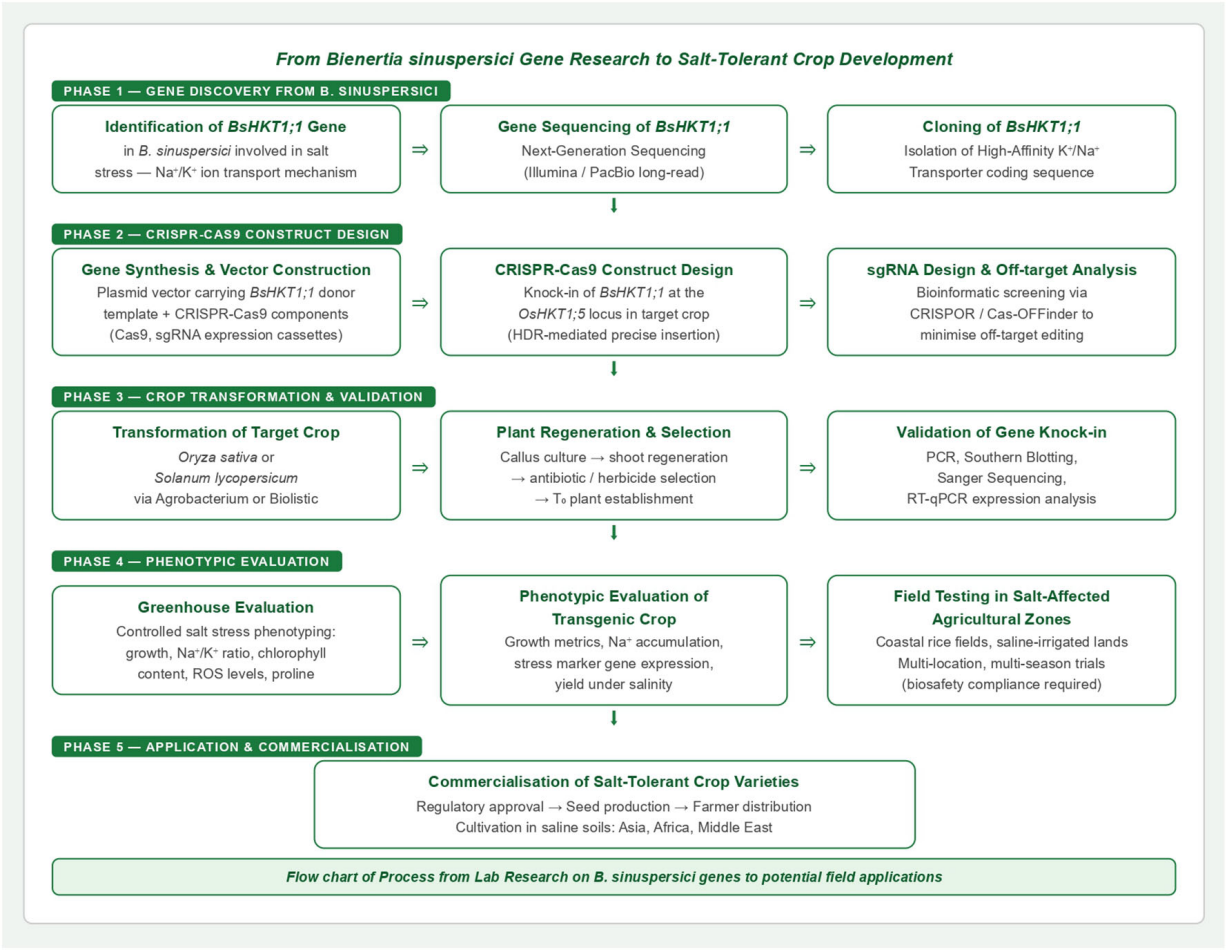
Flow chart process from lab research on *B. sinuspersici* genes to potential field applications.

The identification and understanding of *B. sinuspersici* from molecular phylogenetics and other evolutionary studies has certain importance to crop development and enhancement especially for saline and arid regions of South Asia. **Figure 5** Presents process from lab research on *B. sinuspersici* genes to potential field applications.

### 6.1 Key stress-tolerance genes and their transfer potential

Three classes of *B. sinuspersici* genes are now translation-ready: ion-transport genes (BsHKT1 paralogs, BsSOS1, BsNHX1) for salinity tolerance, osmolyte-biosynthesis genes (BsP5CS, BsBADH) for combined drought–salt resilience, and stress-responsive transcription factors such as BsSERF-1 (Ismail and Horie, 2017; Mickelbart et al., 2015). Direct transgenic transfer has been validated in Brassica rapa and represents the most rapid pathway to crop deployment.

### 6.2 Marker-assisted selection

Once SNPs in *B. sinuspersici* salt-tolerance loci are characterised, marker-assisted selection (MAS) can introgress these alleles into elite South Asian rice and wheat backgrounds via interspecific hybridisation with closely related Amaranthaceae crops such as quinoa and amaranth (Hasan et al., 2021). MAS offers a regulatory-friendly alternative to transgenesis, particularly for markets where genetically modified crops face restrictions.

### 6.3 CRISPR-Cas9 and transgenic crop development

CRISPR-Cas9 editing enables direct precision modification of crop orthologues to mimic *B. sinuspersici* alleles. Targeted base editing of OsHKT1 selectivity-filter residues, or knock-in of halophyte-specific autoinhibitory sequences in OsSOS1, are now technically feasible (Razzaq et al., 2019; Zafar et al., 2020). The chromosome-level genome assembly provides the reference sequence required to design these edits with confidence.

### 6.4 Accelerated domestication as an improvement pathway

A more transformative approach is *de novo* domestication: rather than transferring genes into existing crops, *B. sinuspersici* itself can be domesticated through CRISPR-based modification of seed-shattering, dormancy, and architecture loci (Lemmon et al., 2018; Zsögön et al., 2018). Successful precedents in tomato and groundcherry suggest that a salt-tolerant SCC4 “designer crop” derived from *B. sinuspersici* could be developed within a single decade (Fernie and Yan, 2019).

## 7 Research gaps and future research directions

Despite the resources now available, three critical knowledge gaps limit translational deployment of *B. sinuspersici* alleles.

### 7.1 Gene regulatory networks — incomplete understanding

The transcriptional networks coordinating *B. sinuspersici* SCC4 development and salinity response remain largely uncharacterised. Single-cell RNA sequencing, ATAC-seq, and ChIP-seq applied to chlorenchyma cells across developmental stages and stress conditions are needed to identify the master transcription factors that orchestrate intracellular compartmentalisation (Yang et al., 2021).

### 7.2 Need for multi-year field trials

All current *B. sinuspersici* -derived transgenic validation has been conducted under controlled greenhouse conditions. Multi-year, multi-location field trials in salt-affected South Asian sites particularly the Indus Basin, the Sundarbans coastal belt, and the Khulna region of Bangladesh are essential to confirm that laboratory salt-tolerance gains translate into yield stability under real soil-salinity gradients (Shrivastava and Kumar, 2015; Tester and Langridge, 2010).

### 7.3 Underutilization in south Asian breeding programs

Despite the agronomic relevance of *B. sinuspersici* to the region, no public-sector breeding programme in India, Pakistan, or Bangladesh currently incorporates the species as a genetic donor. Capacity-building in halophyte germplasm collection, regional genome resequencing, and integration with national wheat and rice breeding pipelines is urgently required to translate this resource into food-security outcomes (Hussain et al., 2020).

## 8 Conclusion

*Bienertia sinuspersici* is a remarkable evolutionary innovation: a single-cell C4 halophyte whose 34 Myr stem lineage, dimorphic chloroplasts, integrated SOS–NHX–HKT ion-regulation machinery, and chromosome-level genome resource collectively constitute one of the most under-exploited reservoirs of crop-improvement alleles in the global flora. This review has consolidated the molecular phylogenetics, single-cell C4 biology, and genomic resources of the species and has identified a priority set of breeder-relevant genes, residue-level functional substitutions, and translational pathways. Single-cell C4 photosynthesis and favourable traits such as salinity and drought tolerance make *Bienertia sinuspersici* a crop that holds great potential for increasing future crop resistance. By using such techniques as CRISPR-Cas9 and marker-assisted selection for the desired characteristics, its genetic traits of ion regulation efficiency, Osmo protectant synthesis, and high photosynthetic rate can be utilized. The reviewed literature shows that the adaptive success of *B. sinuspersici* is not based on a single trait, but on the integration of multiple biological systems. Its single-cell C_4_ pathway is supported by vacuole-driven cellular architecture, chloroplast positioning, altered energy metabolism, redox regulation, and strong ion-homeostasis machinery. Gene families such as BsHKT1, BsSOS1, BsNHX, BsCHUP1, BsPEPC, BsPPDK, BsCAβ, BsAPE1, and BsNITR2;1 provide important evidence that stress tolerance and photosynthetic specialization are functionally linked in this species. In particular, the expansion and functional validation of BsHKT1;2 highlight the practical value of *B. sinuspersici* as a donor of salt-tolerance alleles for crop improvement. The developed C4 photosynthesis pathway could also increase water use efficiency and productivity of C3 crops by meeting food demands in conditions of limited arable land. The principal challenge is no longer biological discovery but coordinated translation: from sequence to allele, from allele to crop, from crop to field. South Asian agriculture, threatened by climate-driven salinization and water scarcity, stands to benefit substantially if this translation is undertaken with the urgency it deserves.
