# Supplementary material for: Molecular phylogenetics and evolutionary history of Bienertia sinuspersici: implications for crop improvement in South Asia
Source: Front Plant Sci. 2026 Jun 9;17:1822164. doi: 10.3389/fpls.2026.1822164 (PMC13286840; doi:10.3389/fpls.2026.1822164)
Supplement: Supplementary file 1 [file DataSheet1.docx]

**Supplementary Table S1.** List of chloroplast rbcL sequences retrieved from NCBI GenBank and used for phylogenetic analysis. The table includes species names, accession numbers, gene region information, and sequence type. For complete chloroplast genome accessions, the rbcL coding region was extracted prior to alignment and tree construction.

| **No.** | **Species** | **GenBank Accession** | **Gene Region** | **Sequence Type** | **Database** |
| --- | --- | --- | --- | --- | --- |
| 1 | *Bienertia sinuspersici* | OQ354385 | rbcL | Coding sequence (CDS) | NCBI GenBank |
| 2 | *Antiaris toxicaria* | PP239384 | rbcL | Gene sequence | NCBI GenBank |
| 3 | *Salicornia bigelovii* | PV151546 | rbcL | Coding sequence (CDS) | NCBI GenBank |
| 4 | *Salicornia brachiata* | KR057186 | rbcL | Gene sequence | NCBI GenBank |
| 5 | *Salicornia europaea* | OL449699 | rbcL | Gene sequence | NCBI GenBank |
| 6 | *Alternanthera philoxeroides* | NC_027226 | rbcL (extracted from complete chloroplast genome) | Plastome-derived | NCBI GenBank |
| 7 | *Alternanthera sessilis* | NC_027224 | rbcL (extracted from complete chloroplast genome) | Plastome-derived | NCBI GenBank |
| 8 | *Beta vulgaris* | GCA_040762635 | rbcL | Gene sequence | NCBI GenBank |


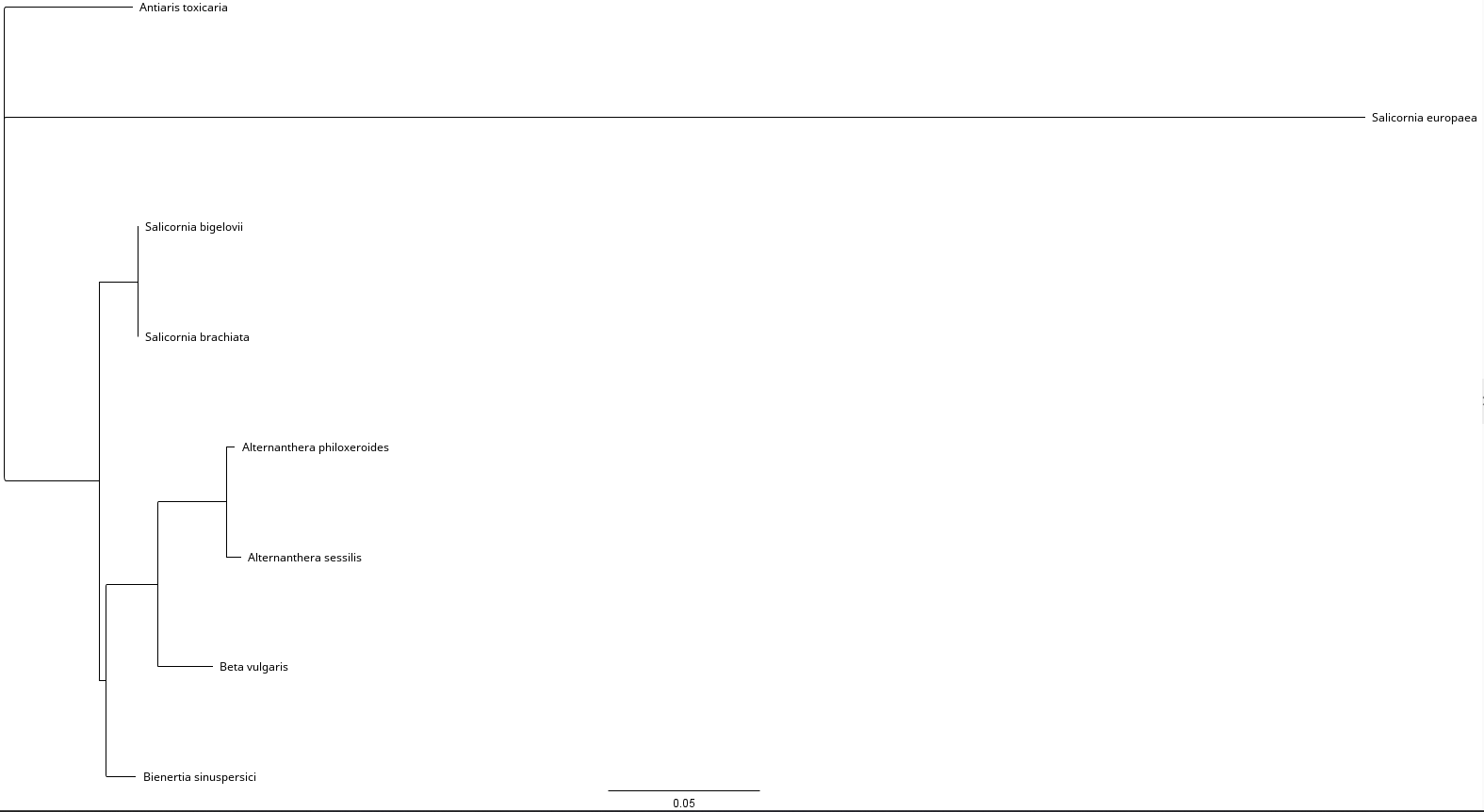


**Supplementary Figure S1.** Maximum-likelihood phylogenetic tree inferred from chloroplast rbcL sequences showing the placement of Bienertia sinuspersici among selected taxa. Branch lengths represent substitutions per site. Node support values are based on 1,000 ultrafast bootstrap replicates. The tree was midpoint-rooted for visualization.


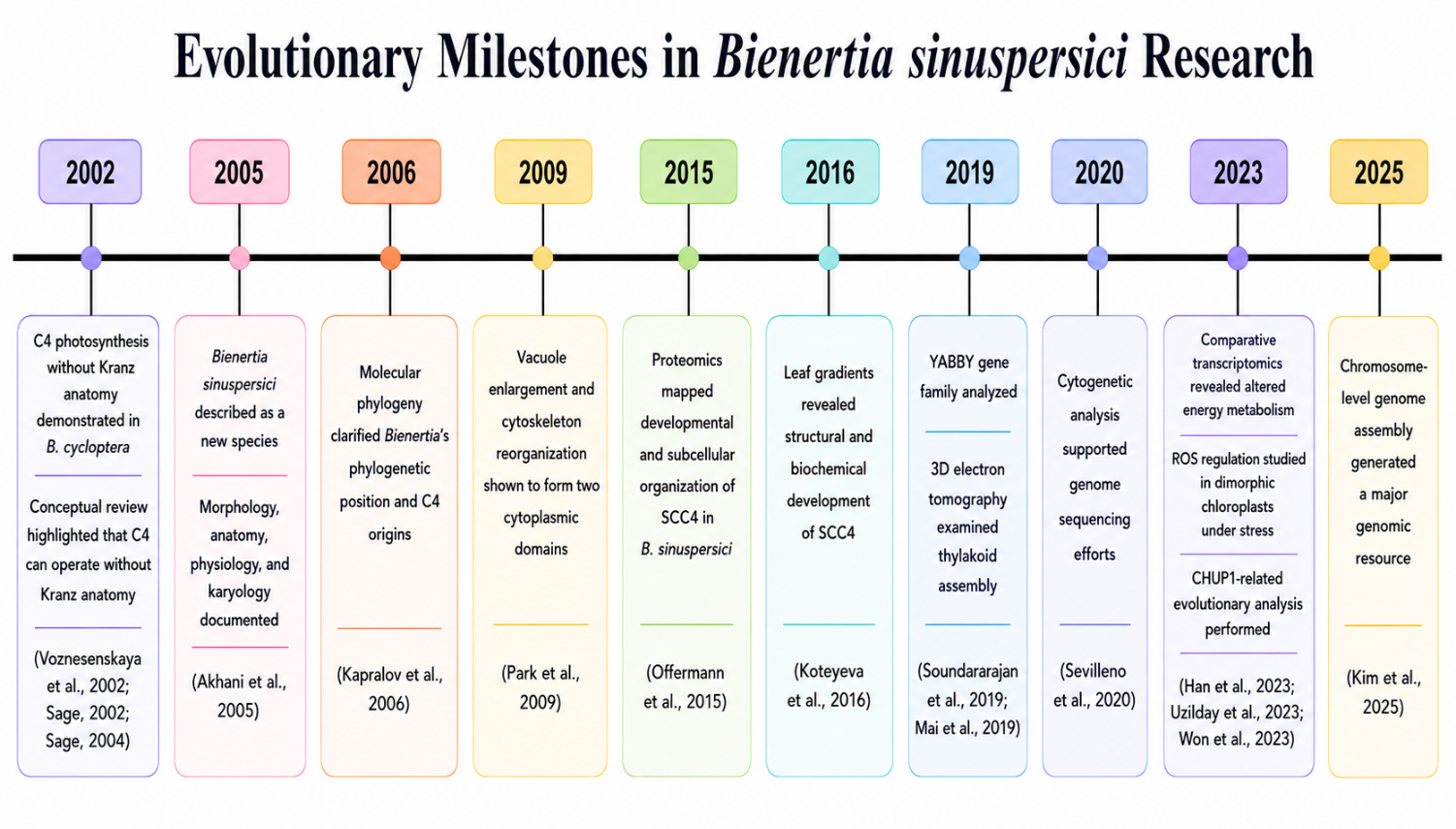
 **Supplementary Figure S2.** Timeline of major research milestones on *Bienertia sinuspersici* and its closest relative *B. cycloptera*, 2002–2025. Key publications are arranged chronologically and indicate the principal evidence type contributed by each study, from the C4 photosynthesis without kranz anatomy (Voznesenskaya, 2002) through anatomical and biochemical characterisation of single-cell C₄ photosynthesis, multi-locus phylogenetic placement, transcriptomic and gene-evolution analyses, to the chromosome-level genome assembly (Kim et al., 2025).

# Comparative Gene Family Analysis: Novel Genes, Alleles, and Pathways in Bienertia sinuspersici

*Compared across Suaeda aralocaspica, Chenopodium quinoa, Arabidopsis thaliana, and major crop orthologs (rice / wheat / maize) — with novelty assessment, breeding potential rating, and primary literature citations*

**Species compared: Bs =** *Bienertia sinuspersici* (focal SCC4 halophyte) | **Sa =** *Suaeda aralocaspica* (related SCC4) | **Cq =** *Chenopodium quinoa* (Amaranthaceae crop) | **At =** *Arabidopsis thaliana* (glycophyte model) | Crops = *Oryza sativa* / *Triticum aestivum* / *Zea mays.*

| **Gene ID in B. sinuspersici** | **Function / Role** | **B. sinuspersici (Copy # / Expression)** | **Suaeda aralocaspica** | **Chenopodium quinoa** | **Arabidopsis thaliana** | **Crop Orthologs (rice / wheat)** | **Novelty of B. sinuspersici Version** | **Breeding / Biotechnology Potential** | **Key Reference(s)** |
| --- | --- | --- | --- | --- | --- | --- | --- | --- | --- |
| BsHKT1;1 BsHKT1;2 BsHKT1;3 | Na⁺ exclusion from transpiration stream; Na⁺/K⁺ ion homeostasis in xylem parenchyma | 3 paralogs (expanded); BsHKT1;2 highest at 100–200 mM NaCl; BsHKT1;3 peaks at ≥200 mM; constitutively higher than AtHKT1;1 | 1–2 copies; less expanded than Bs | 1–2 copies; standard halophyte complement | 1 copy (AtHKT1;1); expression decreases under salt | OsHKT1;5 (rice, Saltol QTL); 8 HKT loci in rice; lower expression under stress than BsHKTs | 3-paralog expansion unique among Amaranthaceae glycophytes; BsHKT1;2 validated in transgenic Brassica rapa higher biomass & root length under salt | Direct allele mining for Na⁺/K⁺ balance in rice & wheat; BsHKT1;2 already functionally validated in transgenic Brassicaceae — high-priority transfer candidate | (Irulappan et al., 2023) |
| BsSOS1 | Plasma-membrane Na⁺ efflux transporter; exports Na⁺ to apoplast via Ca²⁺/SOS2/SOS3 signalling cascade | Single copy; peak expression at 100 mM NaCl; co-regulated with BsHKT1;3 under extreme salinity (200–300 mM); distinct expression kinetics vs. AtSOS1 | Present; functionally characterised | Present; expression documented | AtSOS1; peaks at 200 mM at 2 DAI different trigger point to BsSOS1 | OsSOS1 present; less well-characterised under extreme salinity; salt-sensitive varieties have lower SOS1 activity | BsSOS1 shows distinct concentration-dependent expression threshold (100 mM peak) vs. AtSOS1 (200 mM peak), suggesting a more sensitive salinity sensor allele | Allele with lower salinity detection threshold useful for engineering early-response salt exclusion in crops without fitness penalties at low salinity | (Ali et al., 2021; Irulappan et al., 2023) |
| BsNHX1– BsNHX(n) | Sequesters Na⁺ into vacuoles; maintains cytosolic ion balance; supports turgor under salinity | Expanded family; constitutively high expression even at 0 mM NaCl — suggests pre-adaptive ion management strategy rather than inducible response | Expanded; constitutively active in saline habitat | Present; NHX genes documented; stress-inducible | 6 AtNHX members; stress-inducible rather than constitutive | OsNHX1 (stress-inducible); overexpression improves rice salt tolerance; not constitutively active in elite lines | Constitutive (not inducible) expression profile unique among Amaranthaceae; protective Na⁺ sequestration is active before stress onset, not dependent on stress signalling | Constitutive NHX allele could confer continuous ion protection in saline soils without requiring stress-induction delay; applicable to rice and wheat via MAS or transgenic approach | (Park et al., 2009; Urbanavičiūtė et al., 2021) |
| BsSERF-1 | ERF transcription factor; master regulator of salt and drought stress gene networks; activates multiple downstream stress response pathways | Highly expressed under salt stress; overexpression in transgenic plants improves growth and survival under saline conditions | Presence uncharacterised | Low/uncharacterised | Low/absent in standard conditions; no equivalent characterised ERF with this profile | Low or absent in most elite crop lines; some related ERFs in Triticum aestivum but with different target gene sets | Absent or lowly expressed in most glycophytes and crops; functionally validated via transgenic overexpression directly demonstrated stress tolerance benefit | High-priority candidate for transgenic crop improvement; already demonstrated benefit in overexpression experiments; regulatory elements could be used in stress-inducible promoter constructs | (P. Mehra et al., 2024; Wani et al., 2020) |
| BsFeSOD (PCC- & CCC-forms) | Scavenges reactive oxygen species (ROS) generated by high photosynthetic activity; differential regulation between peripheral (PCC) and central (CCC) chloroplasts under salt/drought | Differentially upregulated in PCC vs. CCC under salt and drought; unique compartment-specific ROS management linked to dimorphic chloroplast system | Similar compartment-specific ROS regulation in SCC4 system | FeSOD present; single-compartment regulation only | FeSOD/MnSOD present; not spatially compartmentalized within single cell | FeSOD present in rice/wheat; upregulated under stress but without intracellular spatial compartmentalization | Compartment-specific FeSOD regulation (PCC vs. CCC) is unique to SCC4 species no equivalent intracellular spatial ROS partitioning exists in any crop or glycophyte model | Source alleles for combined salt + drought + high-light oxidative stress tolerance; the spatial partitioning mechanism informs design of antioxidant engineering strategies in crops | (Uzilday et al., 2023) |
| BsP5CS BsBADH | P5CS: rate-limiting enzyme for proline biosynthesis (osmotic adjustment, membrane protection). BADH: betaine aldehyde dehydrogenase for glycine betaine synthesis (osmotic protection, chaperone function) | Both P5CS and BADH co-expressed at high levels under salt and drought; accumulation of both proline AND glycine betaine simultaneously documented dual osmoprotectant strategy | Both present; similar dual accumulation in SCC4 saline habitat | P5CS present; betaine accumulation documented in quinoa but via distinct pathway | P5CS present and stress-inducible; BADH low / absent Arabidopsis does not synthesize glycine betaine | P5CS in rice/wheat (stress-inducible); BADH absent in rice; maize has low betaine; no crop naturally combines high proline + high betaine | Simultaneous high-level accumulation of BOTH proline and glycine betaine unusual in Amaranthaceae; BADH alleles absent in rice represent a directly transferable gap-filling gene | BADH absent in rice is a direct transfer target; combined proline + betaine overaccumulation could be engineered via stacked gene approach; applicable for both salinity and drought tolerance in rice and wheat | (H. Mehra et al., 2024; Ozturk et al., 2021; Urbanavičiūtė et al., 2021) |
| BsPEPC (PCC-specific isoform) | Initial CO₂ fixation step of C4 cycle; catalyses HCO₃⁻ → oxaloacetate in peripheral chloroplasts (PCC); spatially restricted by unique transit peptide (TP) elements | Constitutively high in PCC of mature leaves (Log2FC ~2.55 vs. young); unique TP mediates PCC-selective import distinct from CCC import and different from all Kranz C4 isoforms | Equivalent C4 PEPC in distal compartment functionally analogous but in proximal/distal rather than peripheral/central organisation | C3 PEPC isoform only; low constitutive expression; no spatial compartmentalization | C3 PEPC only; constitutive, low level; no spatial targeting | C3 PEPC in rice and wheat; no PCC-selective isoform; maize has C4 PEPC but in mesophyll cell (Kranz), not intra-cellular compartment | TP-mediated intracellular PCC-selectivity: first demonstrated selective intra-cellular chloroplast targeting mechanism in any plant; Wimmer et al. (2017) showed these TP elements are transferable to other species | TP sequences could re-engineer PCC-targeted carbon fixation in C3 crops without requiring Kranz anatomy; a potential minimal-gene-set pathway to single-cell C4 rice | (Offermann et al., 2015; Wimmer et al., 2017) |
| BsPPDK (PCC-specific isoform) | Regenerates PEP from pyruvate in PCC; highest Log2FC of all C4 genes (2.85) during C3→C4 leaf maturation; fuels continuous carbon fixation cycle | Highest developmental Log2FC among all C4 genes (Log2FC 2.85); PCC-selective; high stability of PPDK protein even under dark treatment (90% of control) unusual for a light-regulated enzyme | Distal-compartment PPDK; functionally equivalent | PPDK present; expressed constitutively but not C4-associated; no spatial targeting | PPDK present; low level; not C4 associated | PPDK in maize mesophyll (Kranz); rice has PPDK but in standard mesophyll; no intra-cellular compartment targeting | PPDK intra-cellular compartmentation requires specific TP elements not present in Kranz C4 plants; protein stability (90% in dark) suggests enhanced regulatory independence vs. crop PPDKs | TP regulatory sequences for PCC-selective PPDK import are key targets for C4 rice engineering; stable PPDK expression allele useful for engineering continuous C4 activity regardless of light fluctuation | (Han et al., 2023; Lara et al., 2008; Offermann et al., 2015) |
| BsNAD-ME (CCC-specific isoform) | Decarboxylates C4 acids (malate) in central mitochondria/CCC, releasing CO₂ for Rubisco; part of the mitochondria–chloroplast CO₂ shuttle unique to SCC4 | CCC-targeted; co-expressed with mitochondrial transporters (DTC, DIC) and BASS2/NHD1 metabolite shuttles; avgTPMTMM among top expressed C4 genes (4123.15 for NAD-ME2) | Proximal-compartment NAD-ME; functionally analogous in SCC4 | C3 NAD-ME isoform; present but not C4-associated | NAD-ME present as C3 metabolic enzyme; low level; no CCC targeting | NADP-ME (not NAD-ME) in maize mesophyll (NADP-ME type C4); rice has both but neither is C4 decarboxylation-associated; NAD-ME subtype absent as C4 enzyme in crops | CCC-targeted NAD-ME isoform part of coordinated decarboxylation network with metabolite transporters (BASS2, NHD1, DTC, DIC) entire network absent in crops; represents a multi-gene innovation | Entire NAD-ME decarboxylation network (NAD-ME + DTC + DIC + BASS2 + NHD1) is a multi-gene engineering target; reconstituting this network in C3 crops would establish intracellular CO₂ concentration without Kranz anatomy | (Chuong et al., 2006; Han et al., 2023; Park et al., 2009) |
| BsCHUP1 BsCHUP1-like_a BsCHUP1-like_b | Anchors chloroplasts to actin cytoskeleton; controls spatial partitioning of PCC vs. CCC chloroplasts; essential for the vacuole-mediated organelle separation that underlies single-cell C4 | Single CHUP1 copy; CHUP1-like_a and CHUP1-like_b are enlarged in gene size (hidden Markov Model confirmed) compared to all other species; critical for bi-compartment chloroplast organisation | Enlarged CHUP1-like proteins similarly in Suaeda both SCC4 species share this gene size expansion; not present in Kranz C4 | Two CHUP1 copies (allotetraploid duplication); standard gene size; no compartmentalisation role | Single CHUP1; standard size; chloroplast movement only (avoidance response to light) — not compartmentalisation | Single CHUP1 in rice and maize; standard size; no intracellular compartmentalisation role chloroplasts cannot be partitioned to PCC/CCC equivalent | Gene size expansion in CHUP1-like proteins unique to SCC4 species (Bienertia + Suaeda) absent in Kranz C4, C3, and CAM plants; directly controls the vacuole-mediated chloroplast partitioning that is definitional for single-cell C4 | Any engineering of single-cell C4 into crops requires reconstituting CHUP1-like_a and CHUP1-like_b; the enlarged isoforms from Bs are the only known functional templates; priority structural gene for C4 crop engineering programmes | (Park et al., 2009; Won et al., 2023) |
| BsAPE1 BsNITR2;1 | APE1: high-light acclimation protein; stabilises PSII by facilitating grana unstacking — essential for thylakoid remodelling during C3→C4 leaf maturation. NITR2;1: chloroplastic nitrite transporter supporting N-assimilation in the SCC4 energetic context | Both upregulated exclusively at mature-leaf stage in SCC4 species (Log2FC > 1); not upregulated in Kranz C4 species (Amaranthus) — transcriptomically confirmed as SCC4-specific signature genes | Both upregulated — shared SCC4-specific pattern (confirmed in comparative 4-species transcriptomics) | APE1 and NITR2;1 present but not associated with C4 maturation; expression pattern distinct | Both present in A. thaliana; stress-regulated differently; not C4 maturation-associated | Amaranthus hypochondriacus (Kranz C4): NEITHER gene upregulated at maturation — confirming these are SCC4-exclusive, NOT shared by two-cell C4; absent from any crop C4 programme | First transcriptomic identification of genes that distinguish SCC4 from Kranz C4 these define the minimum unique gene set for non-Kranz single-cell C4; no equivalent in any crop or Kranz C4 species | Priority targets for C4 crop engineering: must be included in any SCC4 (non-Kranz) engineering strategy; APE1 and NITR2;1 represent novel, non-crop genes that currently have no MAS or transgenic programmes | (Han et al., 2023; Maeda et al., 2014; Walters et al., 2003) |
| BsCEF genes (PCC-enriched) | Cyclic electron flow (CEF) in PCC generates ATP for C4 CO₂ pumping; linear electron flow (LEF) in CCC generates NADPH for Calvin cycle; spatial separation of CEF vs. LEF within one cell is the energy basis of SCC4 | CEF genes co-expressed with SCC4 cycle genes (functional enrichment analysis); PCC houses CEF (ATP-biased), CCC houses LEF (NADPH-biased); dual energy system operates within a single cell | Similar PCC vs. CCC dual electron flow — shared SCC4 strategy | Standard LEF only; no spatial CEF/LEF compartmentalisation | CEF genes present but not spatially compartmentalised within a single cell | Rice and maize: CEF/LEF not spatially separated within single cells; even in Kranz C4 maize, CEF/LEF separation is between two different cell types (mesophyll/bundle sheath), not within one cell | Intracellular spatial CEF/LEF partitioning driven by dimorphic chloroplast system entirely novel; the co-expression network linking CEF genes to C4 cycle genes in PCC has no equivalent in any sequenced plant genome | Understanding the CEF gene regulatory network is essential for designing any C4 engineering strategy that would avoid Kranz anatomy; improving water-use efficiency in C3 crops through enhanced CEF is a direct target | (Edwards et al., 2004; Han et al., 2023; Offermann et al., 2015) |

**Table Notes and Data Sources**

All gene copy numbers, expression values (Log2FC, avgTPMTMM), and functional characterisations are drawn from primary experimental studies; see References column per row. Blue shading = salt/drought stress tolerance entries. Green shading = C4 photosynthesis and chloroplast biology entries. Gold shading = novelty and breeding potential columns.

*Genome resource: Chromosome-level genome assembly of B. sinuspersici (3,608 Mbp; N50 = 360.8 Mbp; 40,465 annotated genes; BUSCO completeness >95%) is available at Scientific Data (2025). Comparative transcriptomics dataset (4-species: Bs vs. Sa vs. Amaranthus vs. Arabidopsis) deposited with Han et al. (2023), Front. Plant Sci. 14:1202521.*

Abbreviations: SCC4, single-cell C4 photosynthesis; PCC, peripheral compartment chloroplasts; CCC, central compartment chloroplasts; TP, transit peptide; MAS, marker-assisted selection; CEF, cyclic electron flow; LEF, linear electron flow; DAI/WAI, days/weeks after inoculation; Log2FC, log2 fold-change (mature vs. young leaves); avgTPMTMM, average transcripts per million (TMM-normalised).

Ali, A., Raddatz, N., Pardo, J. M., & Yun, D. J. (2021). HKT sodium and potassium transporters in Arabidopsis thaliana and related halophyte species. *Physiologia Plantarum*, *171*(4), 546-558. <https://doi.org/10.1111/ppl.13166>

Chuong, S. D. X., Franceschi, V. R., & Edwards, G. E. (2006). The cytoskeleton maintains organelle partitioning required for single-cell C4 photosynthesis in Chenopodiaceae species. *The Plant Cell*, *18*(9), 2207-2223. <https://doi.org/10.1105/tpc.105.036186>

Edwards, G. E., Franceschi, V. R., & Voznesenskaya, E. V. (2004). Single-cell C4 photosynthesis versus the dual-cell (Kranz) paradigm. *Annual Review of Plant Biology*, *55*, 173-196. <https://doi.org/10.1146/annurev.arplant.55.031903.141725>

Han, S. Y., Kim, W. Y., Kim, J. S., & Hwang, I. (2023). Comparative transcriptomics reveals the role of altered energy metabolism in the establishment of single-cell C4 photosynthesis in Bienertia sinuspersici. *Frontiers in Plant Science*, *14*, 1202521.

Irulappan, V., Park, H. W., Han, S.-Y., Kim, M.-H., & Kim, J. S. (2023). Genome-wide identification of a novel Na+ transporter from Bienertia sinuspersici and overexpression of BsHKT1;2 improved salt tolerance in Brassica rapa. *Frontiers in Plant Science*, *14*, 1302315. <https://doi.org/10.3389/fpls.2023.1302315>

Lara, M. V., Offermann, S., Smith, M., Okita, T. W., Andreo, C. S., & Edwards, G. E. (2008). Leaf development in the single-cell C4 system in Bienertia sinuspersici: expression of genes and peptide levels for C4 metabolism in relation to chlorenchyma structure under different light conditions. *Plant Physiology*, *148*(1), 593-610.

Maeda, S., Konishi, M., Yanagisawa, S., & Omata, T. (2014). Nitrite transport activity of a novel HPP family protein conserved in cyanobacteria and chloroplasts. *Plant and Cell Physiology*, *55*(7), 1311-1324. <https://doi.org/10.1093/pcp/pcu075>

Mehra, H., Yadav, N., Kumar, A., Sawariya, M., Kumar, N., Devi, S., Kumar, S., Dagar, J. C., & Arya, S. S. (2024). Halophytes at the Crossroads: Morphological, Anatomical, Physiological, and Biochemical Responses to Salinity Stress. In *Halophytes vis-à-vis Saline Agriculture: Perspectives and Opportunities for Food Security* (pp. 153-178). Springer.

Mehra, P., Yadav, V., Ramawat, N., Gabdulkhaev, R., Pandey, S., Fatma, S., Abbasi, N., Khurana, J. P., Tuteja, N., Singh, A., & Kumar, S. (2024). Comparative genomic and transcriptomic analysis reveals key genes and regulatory networks involved in salt tolerance of seeds in contrasting rice cultivars. *Frontiers in Plant Science*, *14*, 1271008. <https://doi.org/10.3389/fpls.2023.1271008>

Offermann, S., Friso, G., Doroshenk, K. A., Sun, Q., Sharpe, R. M., Okita, T. W., Wimmer, D., Edwards, G. E., & van Wijk, K. J. (2015). Developmental and subcellular organization of single-cell C4 photosynthesis in Bienertia sinuspersici determined by large-scale proteomics and cDNA assembly from 454 DNA sequencing. *Journal of Proteome Research*, *14*(5), 2090-2108. <https://doi.org/10.1021/pr5011907>

Ozturk, M., Turkyilmaz Unal, B., García-Caparrós, P., Khursheed, A., Gul, A., & Hasanuzzaman, M. (2021). Osmoregulation and its actions during the drought stress in plants. *Physiologia Plantarum*, *172*(2), 1321-1335. <https://doi.org/10.1111/ppl.13297>

Park, J., Knoblauch, M., Okita, T. W., & Edwards, G. E. (2009). Structural changes in the vacuole and cytoskeleton are key to development of the two cytoplasmic domains supporting single-cell C4 photosynthesis in Bienertia sinuspersici. *Planta*, *229*(2), 369-382. <https://doi.org/10.1007/s00425-008-0836-8>

Urbanavičiūtė, I., Bonfiglioli, L., & Pagnotta, M. A. (2021). Salt stress in wheat seedlings: Genome-wide association mapping based on SNP and DArT markers. *Plants*, *10*(7), 1444. <https://doi.org/10.3390/plants10071444>

Uzilday, B., Ozgur, R., Yalcinkaya, T., Sonmez, M. C., & Turkan, I. (2023). Differential regulation of reactive oxygen species in dimorphic chloroplasts of single cell C4 plant Bienertia sinuspersici during drought and salt stress. *Frontiers in Plant Science*, *14*, 1030413. <https://doi.org/10.3389/fpls.2023.1030413>

Walters, R. G., Rogers, J. J. M., Shephard, F., & Horton, P. (2003). Acclimation of Arabidopsis thaliana to the light environment: the role of photoreceptors. *Planta*, *209*(4), 517-527. <https://doi.org/10.1007/s004250050756>

Wani, S. H., Kumar, V., Khare, T., Guddimalli, R., Parveda, M., Solymosi, K., Suprasanna, P., & Kavi Kishor, P. (2020). Engineering salinity tolerance in plants: progress and prospects. *Planta*, *251*(4), 76.

Wimmer, D., Bohnhorst, P., Shekhar, V., Hwang, I., & Offermann, S. (2017). Transit peptide elements mediate selective protein targeting to two different types of chloroplasts in the single-cell C4 species Bienertia sinuspersici. *Scientific Reports*, *7*(1), 41187. <https://doi.org/10.1038/srep41187>

Won, S. Y., Soundararajan, P., Irulappan, V., & Kim, J. S. (2023). In-silico, evolutionary, and functional analysis of CHUP1 and its related proteins in Bienertia sinuspersici-a comparative study across C3, C4, CAM, and SCC4 model plants. *PeerJ*, *11*, e15696. <https://doi.org/10.7717/peerj.15696>
